# Medical mesocosms and cohort differences in victim decisions in spousal violence in Sub-Saharan Africa

**DOI:** 10.3389/fsoc.2025.1266401

**Published:** 2025-08-07

**Authors:** Patricia Elung'ata

**Affiliations:** Department of Sociology, McGill University, Montreal, QC, Canada

**Keywords:** victim behavior, social inequality, gender-based violence, social determinants, health inequalities

## Abstract

**Background:**

Contradictory evidence exists on whether medicine explicates social disparities in health perceptions. This study evaluates healthcare systems as mesocosms to understand social differences in spousal violence perceptions in sub-Saharan Africa, concretely, cohort differences in victim decisions in spousal violence (VDSV).

**Conceptual framework:**

Medical dominance theory criticizes medical power asymmetry, while socio-ecological theory illuminates social disparities in human behavior. This study investigates socio-behavioral patterns in medicine as parallels to spousal violence behaviors, analyzing how power asymmetry influences VDSV in sub-Saharan Africa.

**Methods:**

A cross-sectional study analyzed data from the latest Demographic and Health Surveys (2001–2024) across 31 countries, focusing on 193,232 women aged 15–49 years and their VDSV patterns: none, Discordant, and Other types. Unadjusted odds ratios (ORs) examined associations between VDSV patterns and birth cohorts (BCs) while adjusting for confounders. Interaction terms assessed the impact of neighborhood ethnic diversity, relationship power differences, and healthcare access. A two-level hierarchical multinomial logistic regression model analyzed VDSV variation, considering individual, cluster, and household-level confounders with random country effects. Spatial interpolation addressed geographical clustering. Analyses were performed using the McLogit package in R (Version 4.4.0).

**Results:**

Across BCs, a greater percentage change in predicted marginal probabilities was observed for Other type VDSV compared to Discordant VDSV. Overall, women with recent healthcare access had smaller percentage changes; those with a large relationship power difference, especially, showed the opposite trend. Notably, observed healthcare access effects persisted beyond socioeconomic disparities.

**Discussion:**

Higher VDSV amongst younger birth cohorts suggests lower SV-accepting attitudes. VDSV differences across healthcare access intimate anti-violence intervention exposure effects; relationship power differences play moderating roles. Persistent adjusted healthcare access effects suggest roles for narrowing socio-health inequalities in SSA.

**Conclusion:**

The study results advocate for macro-societal policies within healthcare that address social issues, particularly through theory-based approaches. Future research may evaluate the potential influence of healthcare funding cuts.

## 1 Purpose of study

### 1.1 Medicine and social problems

Evidence indicates that unethical medical practices can exacerbate persistent social issues, like the racial health inequalities highlighted by the controversial Tuskegee syphilis study (Thomas and Quinn, 1991; Gamble, 1997). Conrad (1992) outlines pathways (Conrad, 1992, 2007) for medical social control (Zola, 1972; Illich, 1976), including medical ideology, such as the medical model (Parsons, 1975; Silverman, 1983), medical collaboration, such as workplace screening and intervention for infectious diseases or substance abuse (R. Williams and Williams-Morris, 2000; Marcotte, 2004), incremental population-based medical surveillance (Foucault, 1975; Armstrong, 1995), and the use of medical technology for social control (Conrad and Schneider, 1992; Rose, 2001), such as genetic screening (Rose, 2001; National Research Council (U.S.) et al., 2008).

Some research studies on medical approaches to social problems (Conrad and Schneider, 1992; Ityo, 2010; Dillon, 2020) extend the medical ideology pathway (Conrad, 1992, 2007), proposing macrocosm, mesocosm, and microcosm research analogies. These view research experiment environments as microcosms, explaining larger or equal environments (macrocosm or mesocosm environments) and vice versa (Odum, 1984; Carpenter, 1996). This study examines medicine as a mesocosm to understand social differences in perceptions of spousal violence (SV) in sub-Saharan Africa (SSA). It focuses on cohort differences in medical dominance (MD) victim decisions as mesocosms to understand cohort disparities in spousal violence victim decisions (VDSV).

Research on social aspects of medicine explores social problems at macro-, mezzo-, and micro-levels, their effects on medicine, and vice versa (Evans et al., 1994; Marmot and Wilkinson, 2006), to support evidence-based national policy (Solar and Irwin, 2010). It also examines social problems within medicine and their impacts (Starr, 1982; Conrad, 1992). However, gaps remain in understanding how these interaction effects occur.

### 1.2 Healthcare systems and victim decisions in spousal violence

Surviving SV is partly about victim decisions. VDSV are discussed in various ways, such as whether victims sought help (Ahmad et al., 2009; Itimi et al., 2014; Metheny and Stephenson, 2019), distinguishing between short-term and long-term responses (Kaye et al., 2007; Ahmad et al., 2009), and whether victims utilized attitudinal or emotional responses instead of behavioral responses (Kanagaratnam et al., 2012; Swart, 2013; Arestoff and Djemai, 2016). Some discussions center on barriers to help-seeking, distinguishing between group-level barriers (Rizo and Macy, 2011; Prosman et al., 2014; Huntley et al., 2019; Satyen et al., 2019). Another method contrasts direct and indirect sources of violence (Swart, 2013; Balogun and John-Akinola, 2015; Mannell et al., 2016), such as violence due to victim poverty (Swart, 2013) compared to wealth (Balogun and John-Akinola, 2015). Other studies examine the motivation for victim behavior, whether self-initiated, aka individual agency (Horn et al., 2016; Mannell et al., 2016; McCleary-Sills et al., 2016) or other-initiated, aka structure-initiated (Allen and Devitt, 2012; Horn et al., 2016; Mannell et al., 2016). Regarding other-initiated VDSV, several studies in SSA postulate that SV experiences precede healthcare system contact (Allen and Devitt, 2012; Horn et al., 2016; Mannell et al., 2016); evidence from SSA's high maternal mortality rates, for example, links SV experience among the urban poor to compromised help-seeking during pregnancy and delivery, including mortality-risk exacerbating decisions (Essendi et al., 2011), resulting in higher maternal mortality (Ziraba et al., 2009a; Izugbara and Ngilangwa, 2010). Furthermore, however, evidence suggests that socio-behavioral patterns within medicine may explain those outside medicine and vice versa (Parsons, 1975; Conrad, 2005; Timmermans and Oh, 2010). However, contradictory evidence exists on whether medicine may explain health perceptions, including SV perceptions, while some views disagree (McKeown, 1979; Fogel and Costa, 1997) and others concur (Parsons, 1975; Conrad and Schneider, 1992).

### 1.3 Healthcare systems and social differences in spousal violence perceptions

Generally, social inequalities in health perceptions exist, such as socioeconomic inequalities (Quesnel-Vallee, 2004; Lutfey and Freese, 2005). However, limited evidence exists on social disparities in SV perceptions, including help-seeking behavior (Solar and Irwin, 2010). SV perpetrated by current or former intimate partners is high in SSA, higher among women, and even higher among adolescents (ICF, 2012b; UN-WOMEN, 2024), yet overall, < 50% of victims seek help (ICF, 2012b; UN-WOMEN, 2024). Some victims, supposing SV-accepting family or community social norms, such as protecting family honor (Kanagaratnam et al., 2012; Mannell et al., 2016), ineffectively seek help from family and friends (UN-WOMEN, 2024). Moreover, despite higher SV incidence amongst younger spousal partners, effective responses, including reporting, were less likely, such as with powerful partners (ICF, 2012b; Kenya National Bureau of Statistics et al., 2015). Help-seeking is further unlikely due to the high femicide risk (UN-WOMEN, 2024). The 2024 anti-femicide protests in Kenya, for instance, underscored the exacerbating effect of delayed SV penalties on femicide (Africapractice, 2024). Despite higher female life expectancy estimates worldwide, higher lifetime female disability-life adjusted years estimates are reported for depressive anxiety and headache disorders (Patwardhan et al., 2023), frequently associated with SV (Balogun and John-Akinola, 2015; Hatcher et al., 2022; Metheny et al., 2024). Finally, there is diminished help-seeking, including reporting, due to the intersectionality of disadvantage. Amongst the urban poor, for example (Wilson, 1987; Small and Newman, 2001; African Population and Health Research Center, 2002), environmental degradation exacerbates sexual violence risk and hampers subsequent help-seeking via increased crime and the resulting disruptions in essential service provision disruptions (Corburn and Hildebrand, 2015; Corburn and Sverdlik, 2017).

Contradictory evidence exists on healthcare system approaches to social disparities in health perceptions, including SV perceptions. Some equity perspectives highlight healthcare systems' failure to narrow social inequality. Certain studies indicate incorrect associations between preceding healthcare system improvements and succeeding population health inequality narrowing (Great Britain Working Group on Inequalities in Health et al., 1982; Mackenbach et al., 1997; Lutfey and Freese, 2005). Other studies indicate an incorrect attribution of ongoing population health improvements to current healthcare system enhancements (McKeown, 1979; Fogel and Costa, 1997). Supporting studies, however, link value-free physician–patient interactions (Parsons, 1975) to diminished population health inequalities (Ananth et al., 2001; Alexander et al., 2002; Rittenhouse et al., 2003), whereas value-influenced physician–patient interactions (Abbott, 1988; Conrad, 2005) were associated with persistent health inequalities (Hollingshead and Redlich, 1953; Silverman, 1981, 1983; Solar et al., 2007).

While some SSA studies mention linkages between healthcare systems and SV, limited literature focuses on social differences in SV perceptions. Research in SSA indicates that healthcare systems can integrate health service responses to SV (Colombini et al., 2008, 2017; Joyner and Mash, 2011), enhancing victim support and provider insights. These systems enable comprehensive testing of health service responses to SV (Sprague et al., 2016; Hatcher et al., 2019), leading to improved implementation models (Jacobs and Jewkes, 2002; Joyner and Mash, 2012a). In fact, clinic-tested SV responses were often positively received (Christofides and Jewkes, 2010; Joyner and Mash, 2014). Even basic screening tests effectively identified SV victims among clinic attendees (Christofides and Jewkes, 2010; Joyner and Mash, 2012b). Healthcare systems also help raise community awareness, beyond victim and care provider awareness (Colombini et al., 2008, 2017), and facilitate victim referrals to social services (Colombini et al., 2008, 2017). Furthermore, healthcare systems provide a suitable sampling frame (Prabhu et al., 2011; Delamou et al., 2015; Gibbs et al., 2017) for understanding victims' SV experiences and sociodemographic backgrounds (Hampanda et al., 2014; Hampanda, 2016a,b), although findings may have limited generalizability (Hampanda et al., 2014; Punch, 2014; Hampanda, 2016a). Furthermore, framing SV as a health issue rather than a social one promotes recognition of healthcare's potential impact (Joyner, 2013; Rees et al., 2014a).

Regarding *theoretical limitations*, some SSA studies acknowledge inadequate social theory application, such as impact evaluations, where inadequate socio-contextual information hinders understanding (Colombini et al., 2008; Hatcher et al., 2015; Sprague et al., 2017). Some SSA studies test theories on cross-sectional rather than longitudinal data (Ezeanochie et al., 2011; Prabhu et al., 2011; Delamou et al., 2015; Tusiime et al., 2015; Bernstein et al., 2016; Mahenge et al., 2016; Gibbs et al., 2017; Berhanie et al., 2019), which better captures changes over time (Menard, 2002). Some propose social theories tested on qualitative as opposed to quantitative data (Rees et al., 2014b), others use purposively selected samples (Rees et al., 2014b) instead of representative ones (Shryock et al., 1975), or small data samples (Bernstein et al., 2016; Mahenge et al., 2016; Mohammed et al., 2017), hindering generalizability (Luker, 2008; Greenwell and Salentine, 2018). Other studies do not clearly define their sampling methods (Tusiime et al., 2015), hindering sample validity evaluation (Lee and Forthofer, 2006). Some studies rely on quantitative data without a clear theoretical basis (Ezeanochie et al., 2011; Prabhu et al., 2011; Delamou et al., 2015; Tusiime et al., 2015; Bernstein et al., 2016; Mahenge et al., 2016; Gibbs et al., 2017; Berhanie et al., 2019), other studies propose well-defined theories (Colombini et al., 2008; Joyner and Mash, 2012a) that are not effectively tested, while other studies lacked a named social theory which guided the empirical analysis process (Umeora et al., 2008; Onoh et al., 2013; Delamou et al., 2015).

This study evaluates whether medicine may explicate social differences in SV perceptions in SSA. Hereafter, I define SV perceptions as VDSV, medicine based on the “medical dominance (MD)” theory, and social differences as birth cohort differences. Subsequently, the literature review focuses on *critical debates* surrounding cohort differences in MD victim behavior as mesocosms to elucidate cohort differences in VDSV in SSA. The SSA focus explicates disagreements between health service-based response advocacy (Joyner, 2013; World Health Organization, 2013) and ongoing health disparities (The Africa Health Agenda International Conference Commission et al., 2021). Furthermore, birth cohort differences provide a historical lens (Abramsky et al., 2014; Ezenweke, 2016) to disagreements between high SV rates in low- and middle-income settings, including SSA (World Health Organization, 2013), persistent socio-cultural intervention barriers (Ezenweke, 2016), and various effective time-based behavior change interventions in the region (Abramsky et al., 2014; Kapiga et al., 2019). Additionally, the study examines SV perceptions in SSA, presuming perceptions underlying behavior, particularly where high social desirability bias risk in social normative behavior reporting exists (Mackie et al., 2015; Ezenweke, 2016); furthermore, perceptions can illuminate associated perceptions and related prioritization processes (Porter, 1985; Zedelius et al., 2017).

### 1.4 Background

#### 1.4.1 Mesocosms

According to Odum (1984), mesocosms describe controlled environments where natural behaviors may be observed; in this study, MD illuminates socio-behavioral patterns within medicine, specifically proposing relationship power asymmetry and its effects within medicine (Freidson, 1970; Starr, 1982; Toth, 2015). Bioscience employs mesocosms to compare complex real-world natural systems against bounded and partially enclosed experimental environments (Odum, 1984). In this study, the MD mesocosm explicates socio-behavioral patterns within spousal violence in households in SSA through relationship power asymmetry and its effects (Freidson, 1970; Starr, 1982; Toth, 2015). Parsons proposes teacher–student relationship mesocosms to model physician–patient relationships (Dillon, 2020), while Conrad and Schneider (1992) postulate physician–patient relationship mesocosms for interactions between medical boards and physicians (Conrad and Schneider, 1992); our mesocosm hypothesis proposes comparable interpersonal relationships to elucidate similar social contexts, specifically physician–patient relationship mesocosms of spouse–partner relationships. Across similar social contexts, there are also micro, mezzo, and macro socio-contextual similarities, including analogous cross-system interactions. Furthermore, within similar social contexts, socio-behavioral patterns in interpersonal relationships can elucidate each other; for instance, MD, its socio-contextual influences, and their interactions can explain equivalent interpersonal relationships beyond medicine. Notably, however, equality between the compared environments is assumed (Odum, 1984). Nonetheless, unforeseen inequality may arise for various reasons (Carpenter, 1996); these reasons include environments at different stages of evolution, incomparable environmental boundaries, and unexpected effects of the compared environments on observed social behavior. Generally, however, proof of the viability of medical mesocosms may advance medical SV interventions (Joyner, 2013), surmounting documented challenges within legal redress systems in SSA (Odero et al., 2014; Mannell et al., 2016).

#### 1.4.2 Cohort-related differences and victim decisions

This review examines vital debates surrounding cohort-related differences in MD and SV victim decisions. The limited MD theory in SSA literature suggested a pattern-matched inclusion of related studies, increasing the risk of measurement biases (Trochim, 2005; Punch, 2014). Few hospital-level studies assess intergenerational differences related to MD. Two such studies (Silverman, 1981, 1983) defined generations as parent generations compared to child generations and found differing parent–child responses based on whether the child was below the consenting age. Additionally, parent responses dominated when they differed from children‘s; however, physician opinions prevailed where the physician's view conflicted with either or both parents and children (Silverman, 1981, 1983). Other studies indicate that temporal changes in hospital policy or regulations resulted in shifts in physicians' relationships with MD victims and corresponding victim behavior toward these changes (Weiss and Sutton, 2009; Cooper et al., 2012; Lennan, 2014; Zhou et al., 2019). Several population-level studies suggest that intergenerational differences may explain VDSV in SSA. Some studies evaluate intergenerational differences as variations between the VDSV of parents and their children (Kaye et al., 2007; Kanagaratnam et al., 2012; Decker et al., 2013), birth cohort disparities, or changes across temporal periods (Arestoff and Djemai, 2016; Mannell et al., 2016); at times, these changes were linked to implemented SV interventions (Njuki et al., 2012; Abramsky et al., 2014; Harvey et al., 2018; Naved et al., 2018). Previous debates were somewhat limited (Simister, 2010; Kanagaratnam et al., 2012; Mannell et al., 2016); this study aimed to fill that gap. One expectation from the review is that cohort differences may explain VDSV in SSA, with younger cohorts less accepting of SV (Hypothesis 1).

#### 1.4.3 Medical mesocosms and victim decisions

Below, various debates surrounding medical mesocosm approaches to social disparities in victim decisions are explored, particularly concerning MD theory. MD theory posits that physicians strategically disempower other stakeholders within their field (Freidson, 1970; Starr, 1982); consequently, they gain medical sovereignty (Freidson, 1970; Toth, 2015), technical and professional autonomy (Freidson, 1970; Sandstrom, 2007), and cultural authority (Freidson, 1970; Toth, 2015). Contra-MD arguments highlight that existing research predominantly centers on physicians as perpetrators rather than victims (Freidson, 1970; Starr, 1982). Several reviewed MD studies employed qualitative data, and efforts to derive comparable quantitative data may exacerbate research biases. Finally, autonomous decision-making regarding the mechanisms through which social conditions influence MD (Coburn, 1993, 1999) also increases the risk of measurement biases. Pro-MD arguments recognize a two-way interaction, wherein either the physician or patient can be victims (Gerhardt, 1989), respond uniquely (Silverman, 1981, 1983), with social patterning (Abbott, 1988), but also be value-influenced (Silverman, 1981, 1983; Conrad and Schneider, 1992). The above arguments suggest that MD “mesocosms” may explain cohort differences in VDSV in SSA.

#### 1.4.4 Conceptual framework

Adopting pattern-matching (Trochim, 2005; Punch, 2014) and socioecological theory approaches (Bronfenbrenner, 2005; Mackie et al., 2015), this section proposes how, based on existing evidence, an MD theory (Freidson, 1970; Starr, 1982; Toth, 2015) mesocosm (Odum, 1984) may elucidate cohort differences in VDSV in SSA. Pattern-matching posits hypotheses generation from comparisons of observed and expected conceptual patterns (Trochim, 2005; Punch, 2014); it employs a grounded-theory strategy (Punch, 2014).

##### 1.4.4.1 Socio-ecological theory and cross-system interactions

A study expectation predicated on pattern-matching theory (Trochim, 2005; Punch, 2014) and socio-ecological theory (Bronfenbrenner, 2005) posits that interactions among micro, mezzo, and macro systems may elucidate VDSV in SSA. Mackie et al. (2015) suggest that socio-ecological theory (Bronfenbrenner, 2005) may explain human behavior within SV (specifically across three main sub-systems: microsystems encompassing the conflict arena, the perpetrator (male partner), the victim (female partner), their relationship, and specific SV experiences; mesosystems comprising neighborhoods; and macrosystems incorporating socio-historical factors, such as socioeconomic stratification (Dillon, 2020). Additionally, socio-ecological theory posits that interactions across exosystems (Bronfenbrenner, 2005), such as overarching public policy (Quesnel-Vallée et al., 2021), explain SV.

Despite limitations, some household-based evidence indicates that cross-system interactions, such as socioeconomic inequalities (Wright, 1997; Phelan et al., 2010), explain social disparities in VDSV in SSA. For instance, despite higher SV incidence amongst lower socioeconomic status couples (Itimi et al., 2014), within this group, lower education levels and reduced help-seeking for SV are further associated (Simister, 2010; Bhuwania and Heymann, 2022), sometimes due to government service fee payments (Njuki et al., 2012; Odero et al., 2014). Associations are also reported between MD victim resistance and victim-perpetrator power differences, socioeconomic differences (Sandstrom, 2007; Alubo and Hunduh, 2017), and institutional and government support differences (Coburn, 1999; Alubo and Hunduh, 2017). Notably, however, beyond SV, VDSV emphasizes roles for individual agency (Swart, 2013; Horn et al., 2016), suggesting that SE theory applicability (Assari, 2013; Jefferies, 2016) to VDSV remains unclear.

##### 1.4.4.2 Macrosystem, mesosystem, and microsystem environments

Following pattern-matching theory (Trochim, 2005; Punch, 2014) and socio-ecological theory proposed macrosystems (Mackie et al., 2015), some hospital-based research studies indicate that despite theoretical and methodological limitations, “pre-assigned role change” may explain social differences in MD victim responses (Weiss and Sutton, 2009; Cooper et al., 2012). Pattern-matched (Trochim, 2005; Punch, 2014) household-based studies also indicate that, despite limitations, “pre-assigned social roles” may elucidate social disparities in VDSV in SSA (Kanagaratnam et al., 2012; Gillum et al., 2018). Indeed, structural-functionalism theory (Dillon, 2020), partly characterized by “pre-assigned social roles,” posits that despite theoretical limitations (Parsons, 1975), at the macrosystem level, society dictates accepted victim behavior, gatekeeper behavior, and rules for behavior change through institutional interventions, which vary across social stratification (Dillon, 2020).

Conformable with pattern-matching theory (Trochim, 2005; Punch, 2014) and the socio-ecological theory proposed mesosystems (Mackie et al., 2015), various hospital-based research studies suggest that, notwithstanding theoretical and methodological limitations, the concept of “knowledge exchange across social interactions” may explain social differences in MD victim responses (Wilson et al., 2007; Goldman et al., 2016). Pattern-matched household-based studies (Trochim, 2005; Punch, 2014) indicate roles for “knowledge exchange across social interactions,” such as religious interactions (Swart, 2013; Itimi et al., 2014) in comprehending VDSV social differences, particularly victim resistance planning efforts (Swart, 2013; Kohli et al., 2015). Indeed, symbolic interactionism theory (Goffman, 1974), partly characterized by “knowledge exchange across social interactions,” suggests that despite recognized theoretical limitations (Sandstrom and Kleinman, 2004; Ritzer, 2005; Ritzer and Ryan, 2011), at the mesosystems level, social processes impact the victim's self once or repeatedly depending on whether a fundamental self already exists. However, the effect varies across the corresponding victim reactions, society's reaction to the victims and those like them, and the social processes which connect the two (Goffman, 1974), such as group social interactions.

Finally, according to pattern-matching theory (Trochim, 2005; Punch, 2014) and socio-ecological theory proposed microsystems (Mackie et al., 2015), specific hospital-based evidence suggests that despite theoretical and methodological limitations, “artifactual effects” may elucidate social differences in MD victim responses (Sandstrom, 2007; Ghasi et al., 2020). Additionally, pattern-matched household-based evidence (Trochim, 2005; Punch, 2014) indicates that despite limitations, “artifactual effects” may similarly elucidate VDSV social differences (Arestoff and Djemai, 2016; Kapiga et al., 2019). Furthermore, some proponents of MD theory (Starr, 1982; Toth, 2015) suggest that despite known theoretical limitations (Parsons, 1975), at the microsystems level, “artifactual effects,” such as ineffective bias handling (Sica, 2006; Pannucci and Wilkins, 2010), systematic error biases (Sica, 2006; Morgensten, 2018), and limited reliability and validity of research study approaches (Coggon et al., 2009; Pannucci and Wilkins, 2010), may explain observed MD-related result patterns. As such, a second study expectation, predicated on pattern-matching theory (Trochim, 2005; Punch, 2014) and socio-ecological theory (Mackie et al., 2015) is that cross-system interactions between macrosystem social role change, mesosystem knowledge networks, and microsystem artifactual effects may explain VDSV in SSA (Hypothesis 2).

##### 1.4.4.3 Exosystem environment

Unlike Mackie et al. (2015), Bronfenbrenner (2005)'s version of socio-ecological theory additionally emphasizes roles for exosystems, which include higher-level socio-political determinants, such as changes in governance or overarching public and social policies (Solar et al., 2007; Quesnel-Vallée et al., 2021). Some hospital-based evidence indicates that, despite methodological limitations, national healthcare policy changes can elucidate social disparities in MD victim responses (Coburn, 1993, 1999). Similarly, the Evolutionary Social Change Theory (Dietz et al., 1990), partly characterized by “public policy change,” posits that despite recognized theoretical limitations (Parsons, 1975), at the macrosystems level, victim behavior results partly from co-evolutionary perspectives, integrating both micro-process and macro-process societal change effects on human behavior, including immanent social forces changes, and culture, social organization, and institutional changes (Dietz et al., 1990). In contrast, limited household-based evidence conclusively highlights this same aspect. As such, a third study expectation predicated on pattern-matching theory (Trochim, 2005; Punch, 2014) and socio-ecological theory (Bronfenbrenner, 2005) was that interactions between microsystem artifactual effects, mesosystem social networks, macrosystem social role change, and exosystem healthcare policy may explain VDSV in SSA (Hypothesis 3). Hypothesis 3 also postulates medical mesocosms (Odum, 1984), that MD theory may illuminate socio-behavioral patterns within spousal violence in households in SSA.

## 2 Materials and methods section

### 2.1 Data

The study used demographic and health survey data (DHS), nationally representative household surveys conducted every 5 years across selected low- and middle-income countries. Further information regarding data collection (ICF, 2020), sampling strategy (ICF International, 2012), ethical approval, and other details concerning the study can be found elsewhere (ICF, 2012a; Measure DHS/ICF International, 2013; Croft et al., 2023). Ethical consent was obtained from ICF International, the distributor of these data. Analysis was limited to the most recent country surveys, which included a complete SV module, capturing household-level SV experiences and surrounding circumstances (ICF, 2020); a total of 31 countries in SSA were considered. Survey-specific logical rules and related record-matching facilitated data cleaning and imputation (Gelman and Hill, 2007; Sauro, 2015); fewer than 2% of records were affected. All data cleaning was conducted using Stata 16.0. The final study sample, described in Tables 1–5, comprised 193,232 women aged 15–49 years. A cross-sectional analytic study was carried out to compare three VDSV patterns across birth cohorts while adjusting for confounding factors (Fox, 2016; Oakes and Kaufman, 2017).

**Table 1:** Weighted victim decisions in spousal violence estimates among females aged 15–49 in Sub-Saharan Africa, Latest Country DHS 2003–2024, West Africa.

**Country code**	**Survey year**	**Females 15/49**	**DV Module**	**SV**	**VDSV1**		**VDSV2**		**VDSV3**	
					**Other**	**Discord**	**Other**	**Discord**	**Other**	**Discord**
		***N***	***N***	**Row %**	**Row %**	**Row %**	**Row %**	**Row %**	**Row %**	**Row %**
SSA	2000–24	512,269	193,232	45.5	24.0	21.5	32.3	13.2	31.7	13.8
WA	2000–24	226,170	76,157	50.3	27.0	23.3	37.2	13.2	36.1	14.2
BF	2021	17,659	10,863	53.4	26.7	26.7	46.0	7.5	44.5	9.0
BJ	2017–18	15,928	5,408	51.2	33.0	18.2	39.3	11.9	39.9	11.4
CI	2021	14,877	5,040	35.4	23.8	11.6	26.5	9.0	25.2	10.2
CM	2018	14,677	6,682	44.5	29.8	14.8	30.6	13.9	31.0	13.5
TD	2014–15	17,719	4,283	57.5	11.9	45.6	44.6	12.9	44.7	12.9
GA	2019–21	11,043	4,163	35.2	21.8	13.4	20.8	14.4	18.5	16.6
GH	2022	15,014	5,737	37.5	27.6	9.9	26.9	10.5	25.3	12.2
GM	2019–20	11,865	2,470	42.1	17.3	24.7	29.9	12.2	23.2	18.8
LB	2019–20	8,065	3,120	51.8	28.9	22.9	32.6	19.2	36.8	15.0
ML	2018	10,424	3,784	64.3	10.1	54.2	51.0	13.4	37.7	26.6
NG	2018	38,948	10,678	50.2	32.7	17.5	39.6	10.6	38.6	11.6
SL	2019	16,658	5,248	60.5	27.5	33.0	35.9	24.6	41.0	19.5
ST	2008–09	15,574	1,980	79.5	62.0	17.5	65.2	14.1	67.4	12.1
TG	2013–14	17,719	6,701	41.8	25.4	16.4	31.7	10.1	32.3	9.5

Author calculations using Demographic and Health Surveys (DHS), 2003–2024.

SSA, Sub-Saharan Africa; DV Module, Domestic Violence Module; VDSV, Victim Decisions in Spousal Violence; Discord, Discordant Decisions; Other, Unknown or Concordant Decisions; SV, Spousal Violence Experience Last 12 Months; VDSV1, Spousal Violence Accepting Attitudes despite Spousal Violence Experience; VDSV2, Sought Help for Spousal Violence Despite Spousal Violence Accepting Attitudes; VDSV3, Sought Help for Spousal Violence After Spousal Violence Experience Notwithstanding Attitudes; WA, Western Africa; BF, Burkina Faso; BJ, Benin; CI, Ivory Coast; CM, Cameroon; TD, Chad; GA, Gabon; GH, Ghana; GM, Gambia; LB, Liberia; ML, Mali; NG, Nigeria; SL, Sierra Leone; ST, Sao Tome & Principe; TG, Togo.

**Table 2:** Weighted victim decisions in spousal violence estimates among females aged 15–49 in Sub-Saharan Africa, Latest Country DHS 2003–2024, East Africa.

**Country code**	**Survey year**	**Females 15/49**	**DV Module**	**SV**	**VDSV1**		**VDSV2**		**VDSV3**	
					**Other**	**Discord**	**Other**	**Discord**	**Other**	**Discord**
		***N***	***N***	**Row %**	**Row %**	**Row %**	**Row %**	**Row %**	**Row %**	**Row %**
SSA	2000–24	512,269	193,232	45.5	24.0	21.5	32.3	13.2	31.7	13.8
EA	2000–24	153,955	68,860	44.8	18.4	26.4	30.8	14.0	30.2	14.6
BU	2016–17	17,269	10,188	40.0	11.8	28.2	24.3	15.7	20.9	19.1
CD	2013–14	18,827	6,811	58.8	11.6	47.3	36.7	22.1	36.5	22.3
ET	2016	15,683	5,860	53.6	15.5	38.1	44.7	8.9	37.6	16.0
KE	2022	32,156	16,926	37.7	21.7	16.0	24.7	13.1	26.8	11.0
MD	2021	18,869	7,308	54.2	29.3	24.9	40.8	13.4	40.3	13.9
MR	2019–21	15,714	4,184	27.0	16.0	10.9	22.6	4.4	21.3	5.7
RW	2019–20	13,497	2,788	36.5	16.3	20.2	21.1	15.4	25.9	10.6
TZ	2022	13,266	5,563	42.1	18.5	23.6	29.2	12.9	28.9	13.2
UG	2016	8,674	9,232	53.2	24.6	28.6	32.7	20.5	33.9	19.3

Author calculations using Demographic and Health Surveys (DHS), 2003–2024.

SSA, Sub-Saharan Africa; DV, Domestic Violence; VDSV, Victim Decisions in Spousal Violence; Discord, Discordant Decision; Other, Unknown or Concordant Decision; SV, Spousal Violence Experience Last 12 Months; VDSV1, Spousal Violence Accepting Attitudes despite Spousal Violence Experience; VDSV2, Sought Help for Spousal Violence Despite Spousal Violence Accepting Attitudes; VDSV3, Sought Help for Spousal Violence After Spousal Violence Experience Notwithstanding Attitudes; Discord, Discordant Decision; EA, East Africa; BU, Burundi; CD, Democratic Republic of Congo; ET, Ethiopia; KE, Kenya; MD, Madagascar; MR, Mauritania; RW, Rwanda; TZ, Tanzania; UG, Uganda.

**Table 3:** Weighted victim decisions in spousal violence estimates among females aged 15–49 in Sub-Saharan Africa, Latest Country DHS 2003–2024, Southern Africa.

**Country code**	**Survey year**	**Females 15/49**	**DV Module**	**SV**	**VDSV1**		**VDSV2**		**VDSV3**	
					**Other**	**Discord**	**Other**	**Discord**	**Other**	**Discord**
		***N***	***N***	**Row %**	**Row %**	**Row %**	**Row %**	**Row %**	**Row %**	**Row %**
SSA	2000–24	512,269	193,232	45.5	24.0	21.5	32.3	13.2	31.7	13.8
SA	2000–24	132,144	48,215	38.0	25.5	12.5	26.8	11.2	26.5	11.6
AO	2015–16	14,379	10,519	38.3	25.0	13.3	25.7	12.6	23.4	14.9
LS	2023–24	6,413	2,490	35.5	26.7	8.8	23.7	11.8	23.7	11.8
MZ	2022–23	13,745	4,813	46.1	32.5	13.5	32.8	13.3	30.7	15.4
MW	2015–16	11,698	6,379	54.8	45.5	9.3	38.2	16.6	38.2	16.6
NM	2013	41,821	2,931	21.1	11.8	9.2	19.9	1.2	19.8	1.3
ZA	2016	18,506	4,357	18.1	15.9	2.2	13.6	4.5	14.0	4.0
ZM	2018	16,411	9,503	49.3	22.9	26.4	34.1	15.2	33.6	15.7
ZW	2015	9,171	7,223	41.3	23.9	17.4	26.9	14.4	28.5	12.8

Author calculations using Demographic and Health Surveys (DHS), 2003–2024.

SSA, Sub-Saharan Africa; DV, Domestic Violence; VDSV, Victim Decisions in Spousal Violence; Discord, Discordant Decision; Other, Unknown or Concordant Decision; SV, Spousal Violence Experience; VDSV1, Spousal Violence Accepting Attitudes despite Spousal Violence Experience; VDSV2, Sought Help for Spousal Violence Despite Spousal Violence Accepting Attitudes; VDSV3, Sought Help for Spousal Violence After Spousal Violence Experience Notwithstanding Attitudes; SA, Southern Africa; AO, Angola; LS, Lesotho; MZ, Mozambique; MW, Malawi; NM, Namibia; ZA, South Africa; ZM, Zambia; ZW, Zimbabwe.

### 2.2 Measures

**Figure 1:**
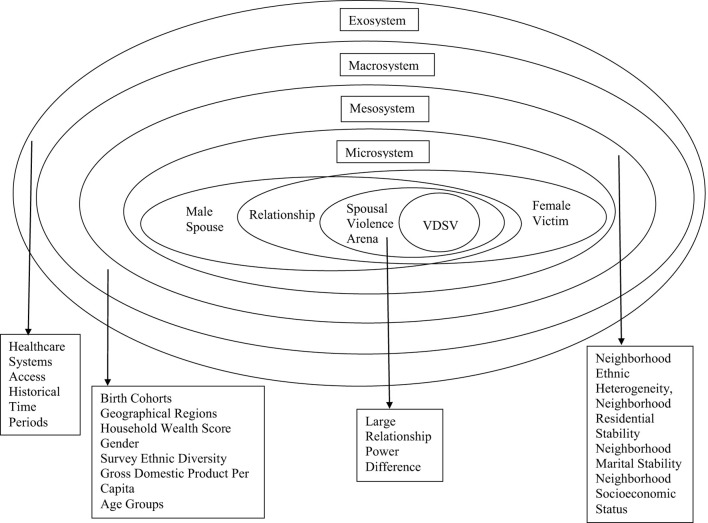
Conceptual framework for female victim decisions in spousal violence (VDSV) in Sub-Saharan Africa.

Refer to Figure 1 for the conceptualization of the primary analytical measures used and the hypothesized relationships between them.

#### 2.2.1 Cross-system interaction measures

##### 2.2.1.1 Dependent variable

*Victim Decisions in Spousal Violence (VDSV):* In line with sick-role theory (Parsons, 1975), the common-sense model of health-related decisions theory (Leventhal et al., 2016), and rational choice theory (Jasso, 2011)—which serve as interpretations of MD theory (Freidson, 1970; Toth, 2015)—I define three measures for VDSV. VDSV patterns 1, 2, and 3 are coded as 0 when respondents reported no spousal violence (0 = None). Additionally, VDSV pattern 1, coded as 2, when respondents express SV-accepting attitudes despite experiencing SV, but not otherwise (1 = Other, including Unknown and Concordant Decisions; 2 = Discordant Decision). In contrast, VDSV pattern 2, coded as 2, was observed when respondents sought help for SV despite expressing SV-accepting attitudes, but not otherwise (1 = Other, including Unknown and Concordant Decisions; 2 = Discordant Decision). VDSV pattern 3, coded as 2, when respondents sought help for SV after experiencing SV, regardless of their attitudes, but not otherwise (1 = Other, including Concordant and Unknown Decisions; 2 = Discordant Decision). Notably, Unknown And Concordant decisions were combined for VDSV patterns 1, 2, and 3 since the focus was on Discordant decisions. Moreover, VDSV patterns 1, 2, and 3 primarily derive from three underlying measures: “SV experience,” “SV accepting attitudes,” and “Sought help for SV.” The binary variable SV experience was coded 1 when respondents experienced SV in the 12 months preceding the survey, whether emotional, sexual, or physical violence, but zero otherwise (Rutstein and Rojas, 2006; Croft et al., 2023). The binary variable SV accepting attitudes takes the value 1 when respondents felt that SV would be warranted in at least one of five hypothetical situations: if she goes out without informing him, neglects the children or argues with him, refuses to have sex with him or burns the food, but zero otherwise (Rutstein and Rojas, 2006; Arestoff and Djemai, 2016; Croft et al., 2023). Finally, the binary variable respondents sought help for SV, was coded 1 when respondents reported seeking help after the SV experience, or they did not seek help but confided in someone about it, but zero otherwise (Rutstein and Rojas, 2006; Croft et al., 2023).

##### 2.2.1.2 Independent variable

**Birth cohorts:** In line with proponents of “Cohort as a Concept in the Study of Social Change” (Ryder, 1965) and “Time, Human Agency and Social Change” (Elder Jr, 1994) as interpretations of structural-functionalism theory (Dillon, 2020), I define birth cohorts. Rather than using survey-reported years of birth, I estimate these as the survey year minus the reported age at survey; this method addresses the issue of surveys spanning more than 1 year (Arestoff and Djemai, 2016). Consequently, for birth years spanning 1947 to 2005, birth cohorts ranging from 1 = 1947–1985 to 2 = 1986+ were defined. Cohort cutoff points were drawn from dates of international family planning policy change (Seltzer, 2002; Garenne, 2018), plus estimated 18-month implementation delays (Ayeni, 2016; Nyarko, 2016; Mutuku, 2023; Adesina, 2024), particularly the 1984 United Nations Mexico City International Conference on Population (United Nations, 1984); proxy measures for less documented national reproductive health policy changes within SSA (Seltzer, 2002; Garenne, 2018); cutoff points assumed equal outcome probability within birth cohorts.

##### 2.2.1.3 Moderating variables

**Neighborhood ethnic diversity (heterogeneity):** In line with the analysis of Social Disorganization Theory (Sampson, 2012; Parks, 2013), as an interpretation of Symbolic Interactionism Theory (Goffman, 1974), I define the first moderating variable as a two-category measure coded (2) when the ethnic diversity within a survey cluster is greater than or equal to the sample grand mean ethnic diversity, and (1) otherwise. Survey clusters constitute neighborhoods. The sample grand mean ethnic diversity is estimated by dividing the total by the count of survey cluster estimates of ethnic diversity. Ethnic diversity is derived from the Entropy Index, which estimates cluster ethnicity distribution; lower scores signify low ethnic diversity, with negative scores viewed as lower than positive scores (Sampson, 2012; Parks, 2013).

**Relationship power-difference:** In line with sick-role theory (Parsons, 1975) as an interpretation of MD theory (Freidson, 1970; Toth, 2015), I define this second moderating variable. This is a derived measure of individual-level microsystem effects, coded (1) when respondents were in a union and were the principal decision-maker in at least one of three household decisions, (2) for respondents not currently in a union, and (0) otherwise (Rutstein and Rojas, 2006; Croft et al., 2023). Relationship power difference was used as a proxy measure for perpetrator behaviors, distinguishing between emotional, sexual, and physical violence solely and the former plus marital control issues (Rutstein and Rojas, 2006; Croft et al., 2023).

**Healthcare system access:** In line with macro-social policy and health inequalities theory (Solar et al., 2007; Quesnel-Vallée et al., 2021) as interpretations of Evolutionary Social Change Theory (Dietz et al., 1990), I define a proxy measure for national healthcare policy changes as the third moderating variable. Access estimates whether respondents sought family planning advice from healthcare professionals, had health insurance coverage, consulted skilled birth attendants for their most recent delivery, or delivered at a healthcare facility. The measure was coded (1) where healthcare access was reported in the 12 months preceding the survey, and (0) otherwise.

#### 2.2.2 Control variables

First, measures to account for unaddressed mesosystem effects.

**Neighborhood marital stability:** Survey clusters constituted neighborhoods. Marital stability within the index survey cluster was calculated as the ratio of the proportion of divorced or separated persons amongst married persons to the proportion of single persons (Sampson, 2012; Parks, 2013).

**Neighborhood residential stability:** a three-category measure, was coded (0) for movers, (1) for non-movers, but (2) otherwise (Emina et al., 2011); survey clusters constituted neighborhoods. Within index households, stay duration was defined as the reported length of stay in years for those coded “96” or “visitor,” but completed age in years for those coded “since birth” or “always.”

**Neighborhood socioeconomic status:** Survey clusters constituted neighborhoods. Cluster Socioeconomic Status was indirectly estimated by calculating the proportion of women aged 15 to 49 years who have attained secondary education or higher (African Population and Health Research Center, 2002).

Furthermore, measures accounting for unaddressed macrosystem effects. Age groups, a derived measure of completed age in years at the time of the interview, categorized into 5-year intervals, ranging from 1 = 15–19 years to 7 = 45–49 years. Household Wealth Score, a derived measure of reported household assets and living conditions, was coded from 1 to 5, with 1 representing the poorest and 5 denoting the wealthiest households (Rutstein and Rojas, 2006; Croft et al., 2023). Gross Domestic Product (GDP) Per Capita was derived by country from International Monetary Fund estimates (IMF, 2024) to capture between-country socioeconomic differences (Magadi, 2017; Goodson and Hayes, 2021; IMF, 2024).

**Survey ethnic diversity:** In line with Social Disorganization Theory (Sampson, 2012; Parks, 2013), as an interpretation of Symbolic Interactionism Theory (Goffman, 1974), I define *Survey Ethnic Diversity* which is derived from the Entropy Index. It estimates various ethnic distributions within each country survey; lower scores indicate less ethnic diversity, with negative scores perceived as lower than positive scores (Sampson, 2012; Parks, 2013).

Moreover, measures adjusting for unaddressed exosystem effects. *Historical Time Periods*, a derived three-category variable, was coded (1) for surveys conducted between 1990 and 2000, (2) for 2001 to 2010, and (3) for 2011 to 2024, aligned with the ‘Time, Human Agency and Social Change' Theory (Elder Jr, 1994), providing interpretations of structural-functionalism theory (Dillon, 2020), and serving as a proxy measure for changes across DHS Data Collection Phases (Rutstein and Rojas, 2006; Croft et al., 2023).

Finally, measures to address unexamined structural-level macrosystem effects. *Geographical Regions*, a classification derived from the countries where interviews were conducted, coded 1 for East, 2 for South, and 3 for West Africa.

### 2.3 Empirical analysis

#### 2.3.1 Descriptive analysis

Each categorical variable was assessed for zero cell values, and recategorizations were implemented where necessary (Lewis-Beck, 1995). For every variable, central tendency and dispersion were estimated using means and standard deviations, respectively (Lewis-Beck, 1995; Lewis-Beck and Lewis-Beck, 2016). For continuous variables, histograms and skewness statistics were employed to evaluate the normality of the variable distribution (Lewis-Beck, 1995; Fox, 2016; Lewis-Beck and Lewis-Beck, 2016); normality implied that means were equal to modes and medians. Regarding skewness statistics, results with *p*-value ≥ 0.05 indicated normality assumptions could be rejected and suitable transformations could be applied (Lewis-Beck, 1995); all such continuous variables were converted into categorical variables (Lee and Forthofer, 2006). All descriptive analyses were conducted using STATA 16.0; see Tables 1–5.

#### 2.3.2 Multivariate analysis

The multivariate analysis sample was restricted to the most recent 2001–2024 country DHS data, focusing on 193,232 women aged 15–49 years. Unadjusted Odds Ratios (ORs) were employed to evaluate the strength of the bivariate association between the dependent variables, VDSV patterns 1–3, and the primary explanatory variable, birth cohorts (Liebetrau, 1983; Lewis-Beck and Lewis-Beck, 2016). The moderating effects of neighborhood ethnic heterogeneity, relationship power difference, and healthcare systems accessibility were subsequently evaluated through interaction terms added to the base models. Control measures, such as household wealth and age groups, were also included in the models to account for potential confounding effects. Analyses were based on cross-sectional data; thus, the results reflect neither a causal association nor the direction of the association (Liebetrau, 1983).

A two-level hierarchical multinomial logistic regression model (Gelman and Hill, 2007; Luke, 2020; Rabe-Hesketh and Skrondal, 2022) was fitted to assess VDSV variation across birth cohorts while controlling for potential confounding effects from social-theory-derived determinants at the individual, cluster, and household levels as fixed effects (level 1), and for country effects as random effects (level 2). Within countries, household-level clustering was comparably limited within the sample (Tables 4, 5), but spatial interpolation was employed to account for clustering at the geographical cluster level (Galster, 2012; Duncan and Kawachi, 2018). Across countries, cluster-level and household-level effects helped to account for the hierarchical structure and clustering within DHS datasets (ICF International, 2012; Greenwell and Salentine, 2018). At the country level, random effects accommodate the possibility that, given the average values of all other independent variables, the probability of the outcome varies due to clustering (Gelman and Hill, 2007; Luke, 2020; Rabe-Hesketh and Skrondal, 2022). However, spatial interpolation effects account for the possibility that even within countries, the likelihood of the outcome additionally varies across geographical clusters (Galster, 2012; Duncan and Kawachi, 2018). All multivariate analyses were performed using the mblogit function in the mclogit package (Elff, 2022) of the R programming language version 4.4.0 (R Development Core Team, 2025), which adjusts for survey weights and design by incorporating sampling weights in the likelihood function (Elff, 2022).

**Table 4:** Spousal violence experience estimates among females aged 15–49 in Sub-Saharan Africa by selected predictors, Latest Country Demographic and Health Surveys, 2003–2024.

**Level**	**Predictors**	**Mean**	**S.D**.	***N***
**No experience and some spousal violence experience**				
Survey	Survey ethnic diversity (−10.3,1)	−1.2	4.0	31
Cluster	Cluster ethnic diversity (−10.1,1)	−0.8	2.4	18,769
	Marital instability (0,30)	0.9	1.1	18,769
	Proportion SECONDARY Plus (0,1)	0.4	0.3	18,769
Household	Household Wealth (1,5)	3.0	1.4	193,232
	Urban–rural residence (0,1)	0.4	0.5	193,232
Individual	Birth cohort (0,1)	0.6	0.5	193,232
	Time period (1,2)	2.0	0.2	193,232
	Healthcare past 12 months (1,2)	1.5	0.5	193,232
	Relationship power–differences (1,3)	2.1	0.7	193,232
	Age group (1,7)	3.5	1.8	193,232
	Residential stability (0,2)	0.7	0.7	193,232
**No spousal Violence experience**				
Survey	Survey ethnic diversity (−10.3,1)	−1.0	3.6	16
Cluster	Cluster ethnic diversity (−10.1,1)	−0.7	2.4	9,903
	Marital instability (0,30)	1.0	1.4	9,903
	Proportion secondary plus (0,1)	0.5	0.3	9,903
Household	Household wealth (1,5)	3.1	1.4	97,237
	Urban–rural residence (0,1)	0.4	0.5	97,237
Individual	Birth cohort (0,1)	0.7	0.5	97,237
	Time period (1,2)	2.0	0.2	97,237
	Healthcare past 12 months (1,2)	1.4	0.5	97,237
	Relationship power–differences (1,3)	2.3	0.8	97,237
	Age group (1,7)	3.1	1.9	97,237
	Residential stability (0,2)	0.6	0.7	97,237
**Some spousal violence experience**				
Survey	Survey ethnic diversity (−10.3,1)	−1.5	4.4	15
Cluster	Cluster ethnic diversity (−10.1,1)	−0.9	2.5	8,866
	Marital instability (0,30)	0.7	0.7	8,866
	Proportion secondary plus (0,1)	0.4	0.3	8,866
Household	Household wealth (1,5)	2.8	1.4	95,995
	Urban–rural residence (0,1)	0.3	0.5	95,995
Individual	Birth cohort (0,1)	0.5	0.5	95,995
	Time period (1,2)	2.0	0.2	95,995
	Healthcare past 12 months (1,2)	1.5	0.5	95,995
	Relationship power–differences (1,3)	1.8	0.6	95,995
	Age group (1,7)	3.9	1.7	95,995
	Residential stability (0,2)	0.7	0.7	95,995

SSA, Sub-Saharan Africa; SV, Spousal Violence; S.D., Standard Deviation; N, Number of Observations.

**Table 5:** Victim decisions in spousal violence estimates in Sub-Saharan Africa among females aged 15–49, Latest Country Demographic and Health Surveys, 2003–2024.

**Level**	**Predictors**	**Mean**	**S.D**.	**N**	**Mean**	**S.D**.	***N***
		**Discordant**			**Other**		
**VDSV3**							
Survey	Survey ethnic diversity (−10.3,1)	−0.6	3.6	9	−2.7	4.8	11
Cluster	Cluster ethnic diversity (−10.1,1)	−0.9	2.3	2,340	−0.9	2.6	5,291
	Marital instability (0,30)	0.6	0.5	2,340	0.6	0.7	5,291
	Proportion secondary plus (0,1)	0.4	0.3	2,340	0.4	0.3	5,291
Household	Household wealth (1,5)	2.8	1.4	29,132	2.8	1.4	66,863
	Urban-rural residence (0,1)	0.3	0.5	29,132	0.3	0.5	66,863
Individual	Birth cohort (0,1)	0.5	0.5	29,132	0.5	0.5	66,863
	Time period (1,2)	2.0	0.2	29,132	1.9	0.2	66,863
	Healthcare past 12 months (1,2)	1.6	0.5	29,132	1.5	0.5	66,863
	Relationship power–differences (1,3)	1.8	0.6	29,132	1.8	0.6	66,863
	Age group (1,7)	3.9	1.7	29,132	3.9	1.7	66,863
	Residential stability (0,2)	0.7	0.6	29,132	0.7	0.7	66,863
**VDSV2**							
Survey	Survey ethnic diversity (−10.3,1)	−0.5	3.4	10	−2.9	5.0	10
Cluster	Cluster ethnic diversity (−10.1,1)	−0.8	2.2	2,252	−1.0	2.6	5,379
	Marital instability (0,30)	0.7	0.6	2,252	0.6	0.7	5,379
	Proportion secondary plus (0,1)	0.4	0.3	2,252	0.3	0.3	5,379
Household	Household wealth (1,5)	2.8	1.4	27,619	2.8	1.4	68,369
	Urban–rural residence (0,1)	0.3	0.5	27,619	0.3	0.5	68,369
Individual	Birth cohort (0,1)	0.5	0.5	27,619	0.5	0.5	68,369
	Time period (1,2)	2.0	0.2	27,619	1.9	0.2	68,369
	Healthcare past 12 months (1,2)	1.6	0.5	27,619	1.5	0.5	68,369
	Relationship power–differences (1,3)	1.8	0.7	27,619	1.8	0.6	68,369
	Age group (1,7)	4.0	1.6	27,619	3.9	1.7	68,369
	Residential stability (0,2)	0.7	0.6	27,619	0.7	0.7	68,369
**VDSV1**							
Survey	Survey ethnic diversity (−10.3,1)	−1.7	4.2	6	−1.7	4.5	14
Cluster	Cluster ethnic diversity (−10.1,1)	−0.9	2.5	3,510	−0.9	2.5	4,121
	Marital instability (0,30)	0.6	0.6	3,510	0.7	0.7	4,121
	Proportion secondary plus (0,1)	0.3	0.3	3,510	0.4	0.3	4,121
Household	Household wealth (1,5)	2.6	1.3	45,761	3.0	1.4	50,234
	Urban–rural residence (0,1)	0.3	0.4	45,761	0.4	0.5	50,234
Individual	Birth cohort (0,1)	0.5	0.5	45,761	0.5	0.5	50,234
	Time period (1,2)	1.9	0.2	45,761	2.0	0.2	50,234
	Healthcare past 12 months (1,2)	1.5	0.5	45,761	1.6	0.5	50,234
	Relationship power–differences (1,3)	1.8	0.6	45,761	1.8	0.7	50,234
	Age group (1,7)	3.9	1.7	45,761	4.0	1.7	50,234
	Residential stability (0,2)	0.8	0.7	45,761	0.6	0.6	50,234

S.D., Standard Deviation; N, Number of Observations; VDSV1, Spousal Violence Accepting Attitudes despite Spousal Violence Experience; VDSV2, Sought Help for Spousal Violence Despite Spousal Violence Accepting Attitudes; VDSV3, Sought Help for Spousal Violence After Spousal Violence Experience Notwithstanding Attitudes.

Sample weight use is occasionally debated (Winship and Radbill, 1994; Lee and Forthofer, 2006); however, sampling weights were applied to adjust for non-response and oversampling. *P*-values of ≤ 0.05 were considered statistically significant. Independent variables' standard error (SE) precision and likelihood ratio test-based parameter testing were employed. Ultimately, the reported results utilized adjusted odds ratios (ORs) with 95% confidence intervals (CIs). Maximum likelihood estimation (MLE) was sought at 100 iterations to achieve convergence Ors (Andersen, 2008). In addition to ORs, post-estimation tests were performed to confirm that multivariate models fitted the data better than null models (prtest ≤ 0.05) (Hoffmann, 2004; Fox, 2016), including predicted marginal probabilities (Curtis et al., 1993; Fox, 2016), Wald tests (Hoffmann, 2004; Fox, 2016), Akaike Information Criterion (AIC), and Bayesian Information Criterion (BIC) scores (Hoffmann, 2004), alongside model log-likelihood tests (Hoffmann, 2004). Statistical conclusion validity, evaluating the plausibility of study findings, was assessed through external validity measures (Trochim, 2005). The Relative Index of Inequality (RII) estimates (Sergeant and Firth, 2006; O'Donnell and Wagstaff, 2008; Moreno-Betancur et al., 2015) were used to evaluate final models against relevant social theories (Trochim, 2005), particularly those addressing socioeconomic disparities in healthcare access. Some limitations in the validity assessments were noted (Fleck and Kuhn, 1979; Trochim, 2005; White, 2015).

## 3 Results

### 3.1 Descriptive analysis results

Tables 1–3 highlight regional variations in SV Experience and VDSV rates across Sub-Saharan Africa (SSA). Compared to SV experience rates in SSA (45.5%), SV experience rates are highest in Western Africa (50.3%), followed by Eastern Africa (44.8%) and Southern Africa (38.0%). For VDSV, the highest rates are seen for Other type VDSV2 (31.6%), Other type VDSV3 (31.0%), and Other type VDSV1 (23.6%). Lower rates occur for Discordant VDSV1 (20.7%), Discordant VDSV3 (13.5%), and Discordant VDSV2 (12.8%). Specifically, for the most reported VDSV types, the highest Other type VDSV2 rates are observed in Western Africa (37.2%), Eastern Africa (30.8%), and Southern Africa (26.9%), conversely the highest Discordant VDSV1 rates are observed in Eastern Africa (26.4%), Western Africa (23.3%), and Southern Africa (12.5%). Additionally, regional estimates of women's spousal violence against partners (calculations not shown) reveal the highest rates in Southern Africa (3.82%), followed by Western Africa (3.69%) and Eastern Africa (2.97%), compared to SSA (3.52%).

Table 4 indicates that in the final analytical sample, spousal violence was more commonly reported in survey years characterized by comparatively lower survey ethnic entropy/diversity, lower cluster ethnic entropy/diversity, lower cluster marital instability, and lower cluster proportions of women with secondary education or higher. Additionally, spousal violence victims were of lower socioeconomic status, lower urbanicity, younger birth cohorts, slightly better healthcare system accessibility, less women's final say in household decision-making, and higher residential stability when compared to women not reporting spousal violence.

Table 5 shows that among those reporting spousal violence, notwithstanding VDSV type, Discordant VDSV and Other type VDSV are evenly distributed across pre-1986 and post-1986 birth cohorts. Discordant VDSV1 and VDSV2 typically occur in areas with lower ethnic diversity, while Other VDSV1 and VDSV2 types were found in higher diversity areas; however, both Discordant and Other type VDSV appear equally in VDSV3 contexts. Across relationship power differences, not much variability is observed when comparing Discordant VDSV against Other type VDSV. Regarding healthcare system access, while Discordant VDSV1 and Discordant VDSV2 were more common among women with better access, Discordant VDSV3 is more prevalent among those with less healthcare access.

### 3.2 Multivariate model selection

Table 6 presents potential regression models of cohort disparities in VDSV in SSA. Based on the reviewed MD literature, four models were evaluated (M1–M4); centered on the lowest AICs, lowest BICs, highest positive and lowest negative log-likelihoods, highest degrees of freedom, lowest deviance, and largest difference between the null model (constant-only model) and the current model, M4 was selected. Typically, the best models were selected based on AIC and BIC measures, but other information criteria, such as log-likelihoods and degrees of freedom, deviance, and likelihood ratio tests (LR tests), helped resolve discrepancies among models. While AIC determines the best predictive models and BIC helps select the “true” model amongst multiple possible models, higher degrees of freedom suggest larger sample sizes and, subsequently, better false null hypotheses rejection; typically, the lowest AICs and highest log-likelihoods also went together (Hoffmann, 2004; Fox, 2016).

**Table 6:** Multivariate multinomial logistic regression model selection for birth cohort differences in victim decisions in spousal violence in Sub-Saharan Africa demographic health surveys 2003–2024.

		**df**	**AIC**	**BIC**	**Log-lik. (Full)**	**LRTest**	**Deviance**
**By VDSV1**							
M1	Birth cohorts + other predictors (Mean Values)	43	331,511.7	331,949.6	−165,712.9	97,889.2	331,425.7
M2	Birth cohorts ^*^ cluster ethnic diversity + other predictors (mean values)	47	331,414.3	331,892.8	−165,660.1	97,994.6	331,320.3
M3	Birth cohorts ^*^ cluster ethnic diversity ^*^ relationship power-difference + other predictors (mean values)	63	312,073.8	312,715.3	−155,973.9	117,367.1	311,947.8
M4	Birth cohorts ^*^ cluster ethnic diversity ^*^ relationship power-difference ^*^ healthcare systems access + other predictors (mean values)	87	310,666	311,551.9	−155,246	118,822.9	310,492
N	Sample size (weighted)						195,389.6
**By VDSV2**							
M1	Birth cohorts + other predictors (mean values)	43	325,990.7	326,428.5	−162,952.3	103,410.2	325,904.7
M2	Birth cohorts ^*^ cluster ethnic diversity + other predictors (mean values)	47	325,923.3	326,401.9	−162,914.7	103,485.6	325,829.3
M3	Birth cohorts ^*^ cluster ethnic diversity ^*^ relationship power-difference + other predictors (mean values)	63	306,449	307,090.5	−153,161.5	122,991.9	306,323
M4	Birth cohorts ^*^ cluster ethnic diversity ^*^ relationship power-difference ^*^ healthcare systems access + other predictors (mean values)	87	305,127.7	306,013.6	−152,476.8	124,361.2	304,953.7
N	Sample size (weighted)						195,389.6
**By VDSV3**							
M1	Birth cohorts + other predictors (mean values)	43	328,560.9	328,998.8	−164,237.5	100,839.9	328,474.9
M2	Birth cohorts ^*^ cluster ethnic diversity + other predictors (mean values)	47	328,494	328,972.6	−164,200	100,914.9	328,400
M3	Birth cohorts ^*^ cluster ethnic diversity ^*^ relationship power-difference + other predictors (mean values)	63	309,142	309,783.5	−154,508	120,298.9	309,016
M4	Birth cohorts ^*^ cluster ethnic diversity ^*^ relationship power-difference ^*^ healthcare systems access + other predictors (mean values)	87	307,806.9	308,692.8	−153,816.5	121,682	307,632.9
N	Sample size (weighted)						195,389.6

Author calculations using Demographic and Health Survey Data, 2003–2024.

VDSV1, Spousal Violence Accepting Attitudes despite Spousal Violence Experience; VDSV2, Sought Help For Spousal Violence Despite Spousal Violence Accepting Attitudes; VDSV3, Sought Help For Spousal Violence After Spousal Violence Experience Notwithstanding Attitudes; AIC, Aikake Criteria: lower values are better for prediction; BIC, Bayesian Information Criteria: smaller positive BIC is better and indicates a better model fit; Log lik. , Log Likelihood Full. Model: higher positive and lower negative values indicate better fit; df, Degrees of freedom: higher values are best fitted for predictions and generally mean larger sample sizes. Higher degrees of freedom also mean more power to reject a false null hypothesis and find a statistically significant result. Deviance: Lower deviance values indicate a better model fit. LRTest: The larger the difference between the null model (a constant-only model) and the current model, including predictors, the stronger the evidence that the model is significant.

### 3.3 Multivariate analysis results

Tables 7–9 present odds ratios for VDSV1, VDSV2, and VDSV3 across birth cohorts (BCs), neighborhood ethnic heterogeneity (NE), relationship power differences (PDs), and healthcare systems access (HC), adjusted for other related predictors. Conversely, Tables 10–14 present changes in predicted marginal probabilities for VDSV1, VDSV2, and VDSV3 across birth cohorts (before and after 1986) adjusted for similar factors with other predictors held constant at mean values.

**Table 7:** Estimated odds ratios (or) and confidence intervals (ci) for spousal violence accepting attitudes despite spousal violence experience across birth cohort differences in Sub-Saharan Africa, Latest Country DHS 2003–2024.

**Predictors**	**Categories**	**Discordant VDSV1**		**Other type VDSV1**	
		**OR**	**CI**	**OR**	**CI**
	(Intercept)	1.17	0.55–2.47	2.32	0.94–5.75
Birth cohort	BC2 (*Ref:*BC1)	1.10	0.86–1.42	1.09	0.83–1.43
Cluster ethnic diversity	NE2 (*Ref:*NE1)	0.82	0.74–0.90	0.90	0.81–1.00
Relationship power-difference	PD2 (*Ref:*PD1)	0.72	0.59–0.88	0.95	0.77–1.18
	PD3	0.82	0.66–1.03	0.75	0.58–0.97
Healthcare systems access	HC2 (*Ref:*HC1)	0.89	0.70–1.12	1.40	1.08–1.81
Urban residence	Rural (*Ref:*Urban)	1.10	1.07–1.14	1.03	0.99–1.07
Wealth quintile	Poorer (*Ref*.Poorest)	0.99	0.95–1.03	0.90	0.86–0.94
	Middle	0.95	0.91–0.99	0.80	0.77–0.83
	Richer	0.83	0.79–0.87	0.62	0.59–0.65
	Richest	0.66	0.62–0.69	0.38	0.36–0.40
Historical time period	2013–2024 (*Ref:*2003–2012)	0.36	0.18–0.74	0.34	0.14–0.82
Cluster residential stability	Non–Movers (*Ref:*Movers)	1.13	1.10–1.17	1.13	1.10–1.17
	Others	0.64	0.38–1.07	1.43	0.76–2.68
Age group	20–24(*Ref:*15–19)	2.86	2.70–3.03	2.06	1.95–2.18
	25–29	3.85	3.63–4.08	2.56	2.41–2.70
	30–34	4.38	4.11–4.67	2.85	2.67–3.03
	35–39	4.66	4.32–5.03	3.16	2.92–3.41
	40–44	4.66	4.30–5.05	3.05	2.81–3.32
	45–49	4.97	4.57–5.40	3.18	2.92–3.47
Survey ethnic diversity		1.02	0.98–1.07	1.02	0.96–1.08
GDP per capita		1.00	1.00–1.00	1.00	1.00–1.00
Cluster secondary plus proportion		0.88	0.82–0.95	0.46	0.42–0.50
Cluster marital instability		0.90	0.88–0.92	0.84	0.82–0.87
Interaction term 1	BC2^*^NE2	1.09	0.94–1.26	1.07	0.91–1.25
Interaction term 2	BC2^*^NE2^*^PD2	0.96	0.80–1.15	0.96	0.79–1.16
	BC2^*^NE2^*^PD3	1.59	1.30–1.95	1.68	1.33–2.12
Interaction term 3	BC2^*^NE2^*^PD2^*^HC2	0.91	0.71–1.16	0.74	0.57–0.96
	BC2^*^NE2^*^PD3^*^HC2	0.78	0.58–1.06	0.43	0.31–0.61
Observations		195,389			

Demographic and Health Surveys(DHS), 2003–2024.

Ref., Reference Category; BC1, Pre-1986, BC2, 1986+; NE1, Less than Sample Grand Mean Cluster Ethnic Diversity, NE2, Sample Grand Mean and Greater; PD1, Final Say in Household Decisions, PD2, No Final Say, PD3, Never Married; HC1, No Healthcare Access, HC2, Healthcare Access in Past 12 Months; VDSV1, Spousal Violence Accepting Attitudes Despite Spousal Violence Experience; GDP, Gross Domestic Product; Discord, Discordant Decisions; Other, Unknown or Concordant Decisions.

**Table 8:** Estimated odds ratios (OR) and confidence intervals (CI) for sought help for spousal violence despite accepting attitudes across birth cohort differences in Sub-Saharan Africa, Latest Country DHS 2003–2024.

**Predictors**	**Categories**	**Discordant VDSV2**		**Other type VDSV2**	
		**OR**	**CI**	**OR**	**CI**
	(Intercept)	3.00	1.66–5.45	0.67	0.27–1.64
Birth cohort	BC2 (*Ref:*BC1)	1.13	0.89–1.44	1.06	0.79–1.42
Cluster ethnic Diversity	NE2 (*Ref:*NE1)	0.87	0.80–0.96	0.84	0.75–0.94
Relationship Power-Difference	PD2 (*Ref:*PD1)	0.89	0.74–1.07	0.67	0.53–0.85
	PD3	0.82	0.66–1.02	0.74	0.56–0.97
Healthcare Systems Access	HC2 (*Ref:*HC1)	1.10	0.88–1.38	0.98	0.74–1.28
Urban Residence	Rural (*Ref:*Urban)	1.06	1.02–1.09	1.09	1.04–1.14
Wealth Quintile	Poorer (*Ref*.Poorest)	0.95	0.91–0.98	0.92	0.88–0.97
	Middle	0.87	0.84–0.91	0.85	0.81–0.90
	Richer	0.73	0.70–0.77	0.70	0.66–0.73
	Richest	0.54	0.51–0.56	0.47	0.44–0.50
Historical Time Period	2013–2024 (*Ref:*2003–2012)	0.29	0.16–0.50	0.52	0.22–1.21
Cluster Residential Stability	Non-movers (*Ref:*Movers)	1.09	1.06–1.12	1.28	1.23–1.32
	Others	1.07	0.71–1.60	0.68	0.36–1.26
Age Group	20–24 (*Ref:*15–19)	2.29	2.18–2.41	2.78	2.59–2.99
	25–29	2.89	2.75–3.04	3.86	3.59–4.15
	30–34	3.27	3.09–3.45	4.39	4.05–4.76
	35–39	3.54	3.30–3.78	4.79	4.36–5.26
	40–44	3.57	3.32–3.84	4.46	4.03–4.92
	45–49	3.82	3.54–4.12	4.55	4.10–5.04
Survey Ethnic Diversity		1.02	0.98–1.05	1.04	0.98–1.09
GDP Per Capita		1.00	1.00–1.00	1.00	1.00–1.00
Cluster Secondary Plus Proportion		0.64	0.59–0.68	0.80	0.73–0.88
Cluster Marital Instability		0.87	0.86–0.89	0.89	0.86–0.91
Interaction Term 1	BC2^*^NE2	1.07	0.93–1.23	1.08	0.91–1.28
Interaction Term 2	BC2^*^NE2^*^PD2	0.97	0.82–1.14	0.94	0.76–1.17
	BC2^*^NE2^*^PD3	1.62	1.34–1.97	1.60	1.24–2.07
Interaction term 3	BC2^*^NE2^*^PD2^*^HC2	0.83	0.66–1.04	0.86	0.65–1.15
	BC2^*^NE2^*^PD3^*^HC2	0.64	0.48–0.86	0.60	0.41–0.87
Observations		195389			

Demographic and Health Surveys(DHS), 2003–2024.

Ref., Reference Category; BC1, Pre-1986, BC2, 1986+; NE1, Less than Sample Grand Mean Cluster Ethnic Diversity, NE2, Sample Grand Mean and Greater; PD1, Final Say in Household Decisions, PD2, No Final Say, PD3, Never Married; HC1, No Healthcare Access, HC2, Healthcare Access in Past 12 Months; VDSV2, Sought Help for Spousal Violence despite Accepting Attitudes; GDP, Gross Domestic Product; Discord, Discordant Decisions; Other, Unknown or Concordant Decisions.

**Table 9:** Estimated odds ratios (OR) and confidence intervals (CI) for sought help for spousal violence after spousal violence experience notwithstanding attitudes across birth cohort differences in Sub-Saharan Africa, Latest Country DHS 2003–2024.

**Predictors**	**Categories**	**Discordant VDSV3**		**Other type VDSV3**	
		**OR**	**CI**	**OR**	**CI**
	(Intercept)	2.75	1.47–5.18	0.87	0.37–2.03
Birth Cohort	BC2 (*Ref:*BC1)	1.09	0.86–1.38	1.19	0.88–1.61
Cluster Ethnic Diversity	NE2 (*Ref:*NE1)	0.85	0.78–0.93	0.89	0.80–1.00
Relationship Power-Difference	PD2 (*Ref:*PD1)	0.82	0.68–0.98	0.83	0.66–1.05
	PD3	0.85	0.69–1.06	0.65	0.49–0.87
Healthcare Systems Access	HC2 (*Ref:*HC1)	1.02	0.81–1.28	1.16	0.88–1.52
Urban Residence	Rural (*Ref:*Urban)	1.06	1.03–1.10	1.07	1.03–1.12
Wealth Quintile	Poorer (*Ref*.Poorest)	0.94	0.91–0.98	0.93	0.89–0.97
	Middle	0.86	0.83–0.90	0.87	0.83–0.91
	Richer	0.72	0.69–0.75	0.73	0.69–0.77
	Richest	0.53	0.50–0.55	0.50	0.47–0.53
Historical Time Period	2013–2024 (*Ref:*2003–2012)	0.30	0.16–0.54	0.46	0.20–1.03
Cluster Residential Stability	Non–Movers (*Ref:*Movers)	1.13	1.10–1.16	1.17	1.13–1.21
	Others	1.11	0.72–1.71	0.62	0.35–1.12
Age Group	20–24 (*Ref:*15–19)	2.42	2.31–2.55	2.40	2.24–2.56
	25–29	3.16	3.01–3.32	3.06	2.86–3.28
	30–34	3.61	3.41–3.81	3.40	3.16–3.67
	35–39	3.93	3.67–4.21	3.66	3.34–4.00
	40–44	3.94	3.67–4.24	3.47	3.15–3.82
	45–49	4.06	3.76–4.38	3.89	3.52–4.29
Survey Ethnic Diversity		1.02	0.98–1.06	1.03	0.97–1.08
GDP Per Capita		1.00	1.00–1.00	1.00	1.00–1.00
Cluster Secondary Plus Proportion		0.65	0.60–0.70	0.76	0.69–0.84
Cluster Marital Instability		0.88	0.86–0.89	0.88	0.85–0.91
Interaction Term 1	BC2^*^NE2	1.09	0.95–1.26	1.02	0.86–1.21
Interaction Term 2	BC2^*^NE2^*^PD2	0.95	0.80–1.12	1.00	0.81–1.24
	BC2^*^NE2^*^PD3	1.69	1.39–2.05	1.46	1.12–1.91
Interaction Term 3	BC2^*^NE2^*^PD2^*^HC2	0.86	0.68–1.08	0.78	0.58–1.03
	BC2^*^NE2^*^PD3^*^HC2	0.63	0.48–0.84	0.62	0.42–0.91
Observations		195389			

Demographic and Health Surveys(DHS), 2003–2024.

Ref., Reference Category; BC1, Pre-1986, BC2, 1986+; NE1, Less than Sample Grand Mean Cluster Ethnic Diversity, NE2, Sample Grand Mean and Greater; PD1, Final Say in Household Decisions, PD2, No Final Say, PD3, Never Married; HC1, No Healthcare Access, HC2, Healthcare Access in Past 12 Months; VDSV3 , Sought Help for Spousal Violence after Spousal Violence Experience; GDP, Gross Domestic Product; Discord, Discordant Decisions; Other, Unknown or Concordant Decisions.

**Table 10:** Effects of neighborhood ethnic heterogeneity (NE) on percent change across birth cohorts (BC) in predicted probabilities of victim decisions in spousal violence (VDSV) in Sub-Saharan Africa, Latest Country DHS 2003–2024.

**VDSV**	**Region**	**BC**	**BC/NE1**	**BC/NE2**
Discord VDSV1	Sub-Saharan Africa	0.55	1.45	(0.01)
	Eastern Africa	0.81	1.50	0.36
	Southern Africa	0.53	1.44	(0.06)
	Western Africa	0.29	1.40	(0.33)
Discord VDSV2	Sub-Saharan Africa	(0.11)	0.86	(0.70)
	Eastern Africa	0.04	0.92	(0.50)
	Southern Africa	(0.08)	0.84	(0.67)
	Western Africa	(0.30)	0.83	(0.93)
Discord VDSV3	Sub-Saharan Africa	0.71	2.02	(0.10)
	Eastern Africa	0.83	2.04	0.06
	Southern Africa	0.79	2.02	0.00
	Western Africa	0.51	2.01	(0.36)
Other VDSV1	Sub-Saharan Africa	4.86	2.07	6.73
	Eastern Africa	5.14	2.13	7.13
	Southern Africa	4.85	2.07	6.67
	Western Africa	4.60	2.02	6.38
Other VDSV2	Sub-Saharan Africa	5.14	2.13	7.13
	Eastern Africa	4.03	1.81	5.52
	Southern Africa	3.91	1.74	5.34
	Western Africa	3.68	1.72	5.06
Other VDSV3	Sub-Saharan Africa	5.14	2.13	7.13
	Eastern Africa	3.75	1.42	5.29
	Southern Africa	3.71	1.40	5.23
	Western Africa	3.42	1.39	4.85

Author calculations using Demographic and Health Survey (DHS), 2003–2024.

VDSV1, Spousal Violence Accepting Attitudes despite Spousal Violence Experience; VDSV2, Sought Help for Spousal Violence despite Accepting Attitudes; VDSV3, Sought Help for Spousal Violence after Spousal Violence Experience; NE1, Less than Sample Grand Mean Cluster Ethnic Diversity; NE2, Sample Grand Mean and Greater; Discord=Discordant Decisions; Other, No Decision or Concordant Decisions.

*Do birth cohorts explain VDSV in SSA? To what extent? (Hypothesis 1)*. Table 10 reveals that when holding other predictors constant at mean values, a 1-unit increase in BCs was associated with changes of 0.55%, minus 0.11%, and 0.71% in predicted marginal probabilities of Discordant VDSV1, VDSV2, and VDSV3, respectively; furthermore, changes of 4.86%, 5.14%, and 5.14%, respectively, in Other VDSV1, VDSV2, and VDSV3 types.

*Does neighborhood ethnic heterogeneity moderate birth cohort differences in VDSV? To what extent? (Hypothesis 1)*. In sub-Saharan Africa, Table 10 indicates, while holding other predictors constant at mean values, with every 1-unit increase in BCs and corresponding 1-unit increase in cluster ethnic diversity, 1.45%, 0.86%, and 2.02% changes in predicted marginal probabilities of Discordant VDSV1, VDSV2, and VDSV3 in neighborhoods with ethnic heterogeneity less than sample grand mean, compared to minus 0.01%, minus 0.70%, and minus 0.10% changes in neighborhoods with ethnic heterogeneity equal to or greater than sample grand mean, respectively. Additionally, Table 10 indicates that under similar conditions, neighborhoods with below sample grand mean ethnic heterogeneity experience 2.07%, 2.13%, and 2.13% changes in predicted marginal probabilities of Other VDSV1, VDSV2, and VDSV3 types, compared to 6.73%, 7.13%, and 7.13% changes in neighborhoods with greater ethnic heterogeneity.

*Do relationship power difference and neighborhood ethnic heterogeneity moderate birth cohort differences in VDSV? To what extent? (Hypothesis 2)*. In SSA, Table 11 indicates, while holding other predictors at mean values, with every 1-unit increase in BCs, average changes in Discordant VDSV1 probabilities of 46.2%, 3.7%, and minus 46.9% across relationship power differences, small (PD1), large (PD2), and never-married (PD3), respectively, in cluster ethnic diversity below sample grand mean. In contrast, average changes in Discordant VDSV1 probabilities of 46.1%, 3.4%, and minus 46.6% across relationship power differences, small (PD1), large (PD2), and never-married (PD3), in cluster ethnic diversity greater than or equal to the sample grand mean. Additionally, Other VDSV1 probabilities reflect changes of 40.4%, 7.6%, and minus 40.5% across relationship power differences, small (PD1), large (PD2), and never-married (PD3), in cluster ethnic diversity below sample grand mean, compared to average changes in Other VDSV1 probabilities of 37.9%, 7.3%, and minus 39.1% across relationship power differences, small (PD1), large (PD2), and never-married (PD3), in cluster ethnic diversity greater than or equal to sample grand mean.

**Table 11:** Effects of neighborhood ethnic heterogeneity (NE) and relationship power difference (PD) on percent change across birth cohorts (BC) in predicted probabilities of victim decisions in spousal violence (VDSV) in Sub-Saharan Africa, Latest Country DHS 2003–2024.

**VDSV**	**Region**	**BC /NE1 /PD1**	**BC /NE1 /PD2**	**BC /NE1 /PD3**	**BC /NE2 /PD1**	**BC /NE2 /PD2**	**BC /NE2 /PD3**
Discord VDSV1	Sub-Saharan Africa	46.18	3.74	(46.93)	46.11	3.41	(46.59)
	Eastern Africa	45.52	3.96	(46.84)	47.19	3.71	(47.00)
	Southern Africa	47.59	3.65	(47.20)	48.43	3.48	(47.27)
	Western Africa	45.44	3.61	(46.73)	42.72	3.02	(45.50)
Discord VDSV2	Sub-Saharan Africa	43.31	4.71	(48.23)	37.56	2.69	(44.49)
	Eastern Africa	44.97	4.85	(48.70)	39.87	3.01	(45.11)
	Southern Africa	42.77	4.67	(48.18)	39.09	2.82	(45.17)
	Western Africa	42.17	4.61	(47.82)	33.72	2.23	(43.19)
Discord VSDV3	Sub-Saharan Africa	43.07	5.83	(47.12)	39.95	3.39	(44.70)
	Eastern Africa	44.51	5.93	(47.63)	41.87	3.67	(45.32)
	Southern Africa	42.61	5.80	(47.07)	41.84	3.60	(45.36)
	Western Africa	42.10	5.75	(46.67)	36.14	2.91	(43.42)
Other VDSV1	Sub-Saharan Africa	40.37	7.61	(40.52)	37.88	7.29	(39.07)
	Eastern Africa	39.73	7.84	(40.43)	38.89	7.61	(39.54)
	Southern Africa	41.71	7.52	(40.84)	40.06	7.36	(39.85)
	Western Africa	39.65	7.48	(40.30)	34.67	6.89	(37.83)
Other VDSV2	Sub-Saharan Africa	47.91	5.80	(48.80)	45.39	8.88	(40.95)
	Eastern Africa	49.63	5.94	(49.26)	47.83	9.23	(41.61)
	Southern Africa	47.36	5.76	(48.75)	47.00	9.02	(41.67)
	Western Africa	46.74	5.70	(48.39)	41.33	8.40	(39.57)
Other VDSV3	Sub-Saharan Africa	48.07	5.47	(49.25)	44.41	8.65	(40.81)
	Eastern Africa	49.56	5.58	(49.73)	46.40	8.94	(41.48)
	Southern Africa	47.59	5.45	(49.20)	46.37	8.88	(41.52)
	Western Africa	47.06	5.39	(48.81)	40.48	8.14	(39.44)

Author calculations using Demographic and Health Surveys (DHS), 2003–2024.

VDSV1, Spousal Violence Accepting Attitudes Despite Spousal Violence Experience; VDSV2, Sought Help for Spousal Violence Despite Spousal Accepting Attitudes; VDSV3, Sought Help for Spousal Violence After Spousal Violence Experience; NE1, Less than Sample Grand Cluster Mean Ethnic Diversity; NE2, Sample Grand Mean and Greater Neighborhood Ethnic Diversity; PD1, Final Say in Household Decisions; PD2, No Final Say; PD3, Never Married; Discord, Discordant Decisions; Other, Unknown or Concordant Decisions.

In SSA, Table 11 indicates, while holding other predictors at mean values, with every 1-unit increase in BCs, average changes in Discordant VDSV2 probabilities of 43.3%, 4.7%, and minus 48.2% across relationship power differences, small (PD1), large (PD2), and never-married (PD3), respectively, in cluster ethnic diversity below sample grand mean. In contrast, average changes in Discordant VDSV2 probabilities of 37.6%, 2.7%, and minus 44.5% across relationship power-differences, small (PD1), large (PD2), and never-married (PD3), in cluster ethnic diversity greater than or equal to the sample grand mean. Additionally, Other VDSV2 probabilities reflect changes of 47.9%, 5.8%, and minus 48.8% across relationship power-differences, small (PD1), large (PD2), and never-married (PD3), in cluster ethnic diversity below sample grand mean, compared to average changes in Other VDSV2 probabilities of 45.4%, 8.9%, and minus 41.0% across relationship power-differences, small (PD1), large (PD2), and never-married (PD3), in cluster ethnic diversity greater than or equal to sample grand mean.

In SSA, Table 11 indicates, while holding other predictors at mean values, with every 1-unit increase in BCs, average changes in Discordant VDSV3 probabilities of 43.1%, 5.8%, and minus 47.1% across relationship power differences, small (PD1), large (PD2), and never-married (PD3), respectively, in cluster ethnic diversity below sample grand mean. In contrast, average changes in Discordant VDSV3 probabilities of 40.4%, 3.4%, and minus 44.7% across relationship power differences, small (PD1), large (PD2), and never-married (PD3), in cluster ethnic diversity greater than or equal to the sample grand mean. Additionally, other VDSV3 probabilities reflect changes of 48.1%, 5.5%, and minus 49.3% across relationship power differences, small (PD1), large (PD2), and never-married (PD3), in cluster ethnic diversity below sample grand mean, compared to average changes in Other VDSV3 probabilities of 44.4%, 8.7%, and minus 40.8% across relationship power differences, small (PD1), large (PD2), and never-married (PD3), in cluster ethnic diversity greater than or equal to sample grand mean.

*Do birth cohorts on VDSV effects, varying across relationship power difference and neighborhood ethnic heterogeneity, also differ with healthcare systems' accessibility? To what extent? (Hypothesis 3)*.

#### 3.3.1 Discordant VDSV1, VDSV2, and VSDV3

In SSA, Table 12 shows, across HC1, with other predictors held constant at mean values, for each 1-unit increase in BCs, Discordant VDSV1 probabilities change by 51.9%, minus 4.1%, and minus 63.3% across relationship power differences, small (PD1), large (PD2), and never-married (PD3), respectively, where cluster ethnic diversity is below the sample grand mean. In contrast, with cluster ethnic diversity at or above the grand mean, the changes were 47.4%, minus 4.7%, and minus 60.9%. Additionally, under similar conditions, across HC2, Discordant VDSV1 probabilities change by 41.1%, 11.8%, and minus 25.1% across relationship power differences, small (PD1), large (PD2), and never-married (PD3) where cluster ethnic diversity is below sample grand mean, compared to changes of 45.8%, 11.8%, and minus 28.3% where cluster ethnic diversity at or above the sample grand mean.

**Table 12:** Effect of healthcare systems accessibility (HC) on percent change across birth cohorts (BC) in predicted probabilities of discordant victim decisions in spousal violence (VDSV) in Sub-Saharan Africa adjusted for neighborhood ethnic heterogeneity (NE), relationship power difference (PD), Latest Country DHS 2003–2024.

**VDSV**	**Region**	**HC**	**BC/NE1/ PD1**	**BC/NE1/ PD2**	**BC/NE1/ PD3**	**BC/NE2/PD1**	**BC/NE2/ PD2**	**BC/NE2/ PD3**
Discord VDSV1	Sub-Saharan Africa	HC1	51.90	(4.14)	(63.26)	47.41	(4.72)	(60.90)
	Eastern Africa	HC1	51.87	(3.76)	(63.31)	49.46	(4.45)	(61.41)
	Southern Africa	HC1	53.07	(4.41)	(63.43)	49.66	(4.89)	(61.50)
	Western Africa	HC1	50.76	(4.25)	(63.03)	43.10	(4.82)	(59.80)
	Sub-Saharan Africa	HC2	41.14	11.81	(25.10)	45.82	11.78	(28.32)
	Eastern Africa	HC2	39.71	11.78	(24.76)	45.75	12.09	(28.36)
	Southern Africa	HC2	42.89	11.98	(25.48)	48.29	12.17	(28.94)
	Western Africa	HC2	40.84	11.66	(25.05)	43.43	11.06	(27.67)
Discord VDSV2	Sub-Saharan Africa	HC1	54.82	(5.96)	(72.14)	51.13	(3.27)	(62.62)
	Eastern Africa	HC1	57.59	(5.77)	(72.45)	54.24	(3.11)	(63.21)
	Southern Africa	HC1	53.85	(6.09)	(72.16)	53.21	(3.29)	(63.25)
	Western Africa	HC1	53.02	(6.04)	(71.83)	45.94	(3.40)	(61.41)
	Sub-Saharan Africa	HC2	30.93	16.34	(9.12)	25.05	8.47	(19.63)
	Eastern Africa	HC2	31.59	16.37	(9.60)	26.65	8.97	(20.01)
	Southern Africa	HC2	30.79	16.41	(8.94)	26.07	8.78	(20.07)
	Western Africa	HC2	30.41	16.22	(8.81)	22.42	7.65	(18.82)
Discord VDSV3	Sub-Saharan Africa	HC1	58.86	(3.85)	(71.72)	50.86	(5.78)	(64.51)
	Eastern Africa	HC1	61.06	(3.77)	(72.08)	53.45	(5.61)	(65.05)
	Southern Africa	HC1	58.13	(3.85)	(71.70)	53.36	(5.73)	(65.10)
	Western Africa	HC1	57.40	(3.93)	(71.39)	45.76	(5.99)	(63.38)
	Sub-Saharan Africa	HC2	26.97	16.11	(6.59)	29.86	13.18	(15.69)
	Eastern Africa	HC2	27.73	16.22	(7.04)	31.14	13.56	(16.15)
	Southern Africa	HC2	26.73	16.05	(6.55)	31.18	13.59	(16.12)
	Western Africa	HC2	26.45	16.05	(6.18)	27.26	12.39	(14.80)

Author calculations using Demographic and Health Surveys (DHS), 2003–2024.

VDSV1, Spousal Violence Accepting Attitudes Despite Spousal Violence Experience; VDSV2, Sought Help for Spousal Violence Despite Spousal Accepting Attitudes; VDSV3, Sought Help for Spousal Violence After Spousal Violence Experience; NE1, Less than Sample Grand Mean Cluster Ethnic Diversity; NE2, ; Sample Grand Mean and Greater Neighborhood Ethnic Diversity; na, Not Applicable; HC1, No Healthcare Access; HC2, Healthcare Access in Past 12 Months; PD1, Final Say in Household Decisions; PD2, No Final Say; PD3, Never Married; Discord, Discordant Decision; Other, Unknown or Concordant Decisions.

Furthermore, in SSA, Table 12 indicates, across HC1, while holding other predictors constant at mean values, for each 1-unit increase in BCs, average changes in Discordant VDSV2 probabilities are 54.8%, minus 6.0%, and minus 72.1% across relationship power-differences, small (PD1), large (PD2), and never-married (PD3) respectively, in clusters ethnic heterogeneity below the sample grand mean. In contrast, for cluster ethnic diversity at or above the grand mean, the changes are 51.1%, minus 3.3%, and minus 62.6%. Additionally, across HC2, under similar conditions, the average changes in Discordant VDSV2 probabilities were 30.9%, 16.3%, and minus 9.1% across relationship power differences, small (PD1), large (PD2), and never-married (PD3), respectively, in cluster ethnic diversity below the sample grand mean, compared to 25.1%, 8.5%, and minus 19.6% for cluster ethnic diversity at or above the sample grand mean.

Finally, in SSA, Table 12 indicates, across HC1, with other predictors held constant at mean values, for each 1-unit increase in BCs, average changes in Discordant VDSV3 probabilities are 58.9%, minus 3.9%, and minus 71.7% across relationship power differences, small (PD1), large (PD2), and never-married (PD3), respectively, in clusters ethnic heterogeneity below the sample grand mean. In contrast, for cluster ethnic diversity at or above the grand mean, the changes are 50.9%, minus 5.8%, and minus 64.5%. Additionally, across HC2, under similar conditions, the average changes in Discordant VDSV3 probabilities were 27.0%, 16.1%, and minus 6.6% across relationship power differences, small (PD1), large (PD2), and never-married (PD3), respectively, in cluster ethnic diversity below sample grand mean, compared to 30.0%, 13.2%, and minus 15.7% for cluster ethnic diversity at or above the sample grand mean.

#### 3.3.2 Other type VDSV1, VDSV2, and VSDV3

In SSA, Table 13 shows, with other predictors held constant at mean values, for every 1-unit increase in BCs, average changes in Other VDSV1 type probabilities across HC1 are 59.1%, minus 3.7%, and minus 59.0% across relationship power differences, small (PD1), large (PD2), and never-married (PD3), respectively, in clusters ethnic heterogeneity below the sample grand mean. In contrast, for cluster ethnic diversity at or above the grand mean, the changes are 56.0%, minus 3.2%, and minus 56.7%. Similarly, for HC2, average changes in Other VDSV1 type probabilities are 23.4%, 10.9%, and minus 15.7% across relationship power differences, small (PD1), large (PD2), and never-married (PD3), in cluster ethnic diversity below the sample grand mean, compared to 21.7%, 10.8%, and minus 15.7% for cluster ethnic diversity at or above the sample grand mean.

**Table 13:** Effect of healthcare systems accessibility (HC) on percent change across birth cohorts (BC), in predicted probabilities of other victim decisions in spousal violence (VDSV) in Sub-Saharan Africa adjusted for neighborhood ethnic heterogeneity (NE), and relationship power difference (PD), Latest Country DHS 2003–2024.

**VDSV**	**Region**	**HC**	**BC/NE1/ PD1**	**BC/NE1/ PD2**	**BC/NE1/ PD3**	**BC/NE2/PD1**	**BC/NE2/ PD2**	**BC/NE2/ PD3**
OtherVDSV1	Sub-Saharan Africa	HC1	59.12	3.67	(58.97)	55.97	3.19	(56.66)
	Eastern Africa	HC1	59.09	4.09	(59.03)	58.14	3.48	(57.23)
	Southern Africa	HC1	60.35	3.38	(59.16)	58.35	3.00	(57.32)
	Western Africa	HC1	57.93	3.55	(58.72)	51.41	3.09	(55.44)
	Sub Saharan Africa	HC2	23.42	10.89	(15.73)	21.68	10.75	(15.67)
	Eastern Africa	HC2	22.16	10.87	(15.36)	21.62	11.06	(15.72)
	Southern Africa	HC2	24.95	11.06	(16.16)	23.74	11.14	(16.40)
	Western Africa	HC2	23.15	10.75	(15.68)	19.68	10.04	(14.91)
OtherVDSV2	Sub-Saharan Africa	HC1	69.65	2.08	(70.04)	60.17	4.20	(59.08)
	Eastern Africa	HC1	72.68	2.29	(70.37)	63.46	4.37	(59.73)
	Southern Africa	HC1	68.59	1.95	(70.06)	62.38	4.17	(59.77)
	Western Africa	HC1	67.68	2.00	(69.70)	54.67	4.05	(57.75)
	Sub-Saharan Africa	HC2	26.72	8.82	(17.83)	31.77	13.07	(17.12)
	Eastern Africa	HC2	27.36	8.85	(18.27)	33.46	13.60	(17.51)
	Southern Africa	HC2	26.59	8.90	(17.67)	32.85	13.40	(17.56)
	Western Africa	HC2	26.22	8.72	(17.55)	29.01	12.22	(16.28)
OtherVDSV3	Sub-Saharan Africa	HC1	67.95	1.59	(70.14)	60.68	5.70	(58.07)
	Eastern Africa	HC1	70.27	1.67	(70.52)	63.45	5.89	(58.71)
	Southern Africa	HC1	67.18	1.59	(70.11)	63.35	5.76	(58.77)
	Western Africa	HC1	66.41	1.50	(69.79)	55.25	5.46	(56.73)
	Sub-Saharan Africa	HC2	28.43	8.68	(19.08)	29.52	10.88	(18.87)
	Eastern Africa	HC2	29.20	8.79	(19.47)	30.79	11.25	(19.31)
	Southern Africa	HC2	28.19	8.63	(19.05)	30.83	11.28	(19.29)
	Western Africa	HC2	27.90	8.63	(18.73)	26.92	10.10	(18.01)

Author Calculations using Demographic and Health Surveys (DHS), 2003–2024.

VDSV1, Spousal Violence Accepting Attitudes Despite Spousal Violence Experience; VDSV2, Sought Help for Spousal Violence Despite Accepting Attitudes; VDSV3, Sought Help for Spousal Violence After Violence Experience; NE1, Less than Sample Grand Mean Cluster Ethnic Diversity; NE2, Sample Grand Mean and Greater; na, Not Applicable; HC1, No Access; HC2, Access in Past 12 Months; PD1, Final Say in Household Decisions; PD2, No Final Say; PD3, Never Married; Discord, Discordant Decisions; Other, Unknown or Concordant Decisions.

Furthermore, In SSA, Table 13 indicates, with other predictors held constant at mean values, for each 1-unit increase in BCs, average changes in Other VDSV2 type probabilities across HC1 are 69.7%, 2.1%, and minus 70.0% across relationship power differences, small (PD1), large (PD2), and never-married (PD3), respectively, with cluster ethnic diversity below the sample grand mean. In contrast, for cluster ethnic diversity at or above the grand mean, the changes are 60.2%, 4.2%, and minus 59.1%. Similarly, across HC2, average changes of 26.7%, 8.8%, and minus 17.8% in Other VDSV2 type probabilities are observed across relationship power differences, small (PD1), large (PD2), and never-married (PD3), for cluster ethnic diversity below sample grand mean, compared to 31.8%, 13.1%, and minus 17.1% for cluster ethnic diversity at or above the sample grand mean.

Finally, in SSA, Table 13 indicates, with other predictors held constant at mean values, for each 1-unit increase in BCs, average changes in Other VDSV3 type probabilities across HC1 are 68.0%, 1.6%, and minus 70.1% across relationship power differences, small (PD1), large (PD2), and never-married (PD3), respectively, with cluster ethnic diversity below the sample grand mean. When cluster ethnic diversity is at or above the grand mean, the changes are 60.7%, 5.7%, and minus 58.1%. Furthermore, across HC2, under similar conditions, average changes in Other VDSV3 types probabilities are 28.4%, 8.7%, and minus 19.1% across relationship power differences, small (PD1), large (PD2), and never-married (PD3), in cluster ethnic diversity below sample grand mean, compared to 29.5%, 10.9% and minus 18.9% at or above the sample grand mean.

#### 3.3.3 Statistical conclusion validity assessment

Table 14 presents results of the external validity assessment, including the relative index of inequality (RII) estimates comparing predicted marginal probabilities from final models for the poorest and richest groups (SES1 and SES5). The final model probabilities were those adjusted for BCs, NE, PD, and HC (discussed above). The RII estimates confirm that healthcare access contributes to cohort disparities in VDSV in SSA; smaller poor-rich ratios of Discordant VDSV1, VDSV2, and VDSV3 probabilities for each 1-unit increase in BCs among women reporting healthcare systems access in the 12 months preceding the survey compared to those reporting no access. The above-observed patterns are further supported by smaller poor-rich ratios of Other type VDSV1, VDSV2, and VDSV3 probabilities for each 1-unit increase in BCs changes among women reporting healthcare systems access in the 12 months preceding the survey.

**Table 14:** Socioeconomic differences (SES) in healthcare systems access (HC) effects on percent change across birth cohorts (BC) in predicted probabilities of victim decisions in spousal violence (VDSV) in Sub-Saharan Africa, adjusted for neighborhood ethnic heterogeneity (NE) and relationship power differences (PD), Latest Country DHS 2003–2024.

**VDSV**	**HC**	**RII**	**NE1**	**NE1**	**NE1**	**NE2**	**NE2**	**NE2**
			**PD1**	**PD2**	**PD3**	**PD1**	**PD2**	**PD3**
Discord VDSV1	HC1	SES1	0.61	0.90	0.93	0.62	1.05	0.92
		/SES5						
	HC2	SES1	0.62	0.74	0.63	0.69	0.72	0.87
		/SES5						
Discord VDSV2	HC1	SES1	0.58	0.97	0.93	0.60	1.13	0.92
		/SES5						
	HC2	SES1	0.68	0.78	0.68	0.61	0.65	0.83
		/SES5						
Discord VDSV3	HC1	SES1	0.59	0.98	0.93	0.60	1.08	0.92
		/SES5						
	HC2	SES1	0.65	0.78	0.54	0.65	0.75	0.79
		/SES5						
Other VDSV1	HC1	SES1	0.61	1.14	0.92	0.64	0.98	0.90
		/SES5						
	HC2	SES1	0.71	0.66	0.88	0.57	0.76	0.70
		/SES5						
Other VDSV2	HC1	SES1	0.62	1.10	0.92	0.63	0.90	0.91
		/SES5						
	HC2	SES1	0.64	0.65	0.84	0.67	0.75	0.80
		/SES5						
Other VDSV3	HC1	SES1	0.62	1.04	0.92	0.63	0.92	0.90
		/SES5						
	HC2	SES1	0.66	0.65	0.84	0.65	0.71	0.82
		/SES5						

Author calculations using Demographic and Health Surveys (DHS), 2003–2024.

VDSV1, Spousal Violence Accepting Attitudes Despite Spousal Violence Experience; VDSV2, Sought Help for Spousal Violence Despite Spousal Accepting Attitudes; VDSV3, Sought Help for Spousal Violence After Violence Experience; NE1, Less than Sample Grand Mean Cluster Ethnic Diversity; NE2, Sample Grand Mean and Greater; na, Not Applicable; HC1, No Access; HC2, Access in Past 12 Months; SES5, Richest Wealth Quintile; SES1, Poorest Wealth Quintile; PD1, Final Say in Household Decisions; PD2, No Final Say; PD3, Never Married; Discord, Discordant Decisions; Other, Unknown or Concordant Decisions; RII, Relative Index of Inequality Estimates (SES5/SES1).

## 4 Discussion and conclusion

Contradictory evidence exists on whether medicine may explain social disparities in health perceptions. This study examines whether medicine could explicate social differences, particularly cohort differences in victim decisions in spousal violence (VDSV) in sub-Saharan Africa (SSA); herein, “medicine” serves as a controlled environment or “mesocosm” for observing natural behaviors (Odum, 1984), while VDSV represents spousal violence (SV) perceptions.

Across BCs, a greater percentage change in predicted marginal probabilities for other type VDSV compared to discordant VDSV was observed at mean values. When analyzing neighborhood ethnic heterogeneity, a smaller increase across BCs in predicted marginal probabilities was observed for discordant VDSV compared to other type VDSV in higher cluster ethnic diversity areas (NE2) compared to lower cluster ethnic diversity areas (NE1); observed differences diminished after adjusting for relationship power differences and healthcare system access.

Regarding the overall direction of change across BCs, the findings suggest that across large power difference (PD1), both Discordant and Other type VDSV probabilities increase across BCs; younger cohorts exhibit higher VDSV rates. Across never-married women (PD3), both Discordant and Other type VDSV probabilities decrease across BCs; younger cohorts demonstrate lower VDSV rates. However, across small power difference (PD2), mixed patterns emerged: among those with recent healthcare access (HC2), both Discordant and Other type VDSV predicted marginal probabilities increase across BCs; younger cohorts exhibit greater VDSV rates. However, among those without recent healthcare access (HC1), the probabilities decrease across BCs, which means younger cohorts show lower VDSV rates.

Specifically concerning healthcare access, the following patterns were observed. For large power difference (PD1), for example, women with recent healthcare access (HC2) show about half the percentage change across BCs in Discordant and Other type VDSV-predicted marginal probabilities compared to those without. Similarly, for never-married women (PD3), the change is about a quarter. In contrast, for small power difference (PD2), women with recent healthcare access (HC2) experience a threefold change in Discordant and Other type VDSV-predicted marginal probabilities compared to those without.

Finally, the statistical conclusion validity assessment (Trochim, 2005) using Relative Index of Inequality (RII) estimates (Sergeant and Firth, 2006; O'Donnell and Wagstaff, 2008; Moreno-Betancur et al., 2015) confirms that healthcare systems access modifies socioeconomic health inequalities in the study sample, though some limitations were noted in the validity assessments (Fleck and Kuhn, 1979; White, 2015).

Household-level findings support the medical dominance (MD) theory, which examines social interactions within healthcare systems through the lens of relationship power asymmetry and its effects (Freidson, 1970; Starr, 1982). First, evidence concurs that power-asymmetry relationships, such as those in spouse–partner relationships, identical to physician–patient relationships, are biased across specific social factors (Abbott, 1988; Dillon, 2020); statistical significance for selected social determinants, relationship power differences, and healthcare system accessibility were observed. Moreover, findings challenge the value-free perspective of the sick-role theory (Parsons, 1975; Dillon, 2020), and reveal statistically significant cross-system interaction modification effects for spouse–partner relationships, similar to those in physician–patient relationships; power-asymmetry relationships may be value-influenced (Conrad and Schneider, 1992; Dillon, 2020).

Furthermore, observed cross-systemic interaction effects corroborate macro-societal socio-ecological theories (Bronfenbrenner, 2005; Mackie et al., 2015) by illustrating how structure-constrained human agency can affect VDSV (Archer, 2000; Davidson, 2015); discordant decision-making has been associated with constrained individual agency (Davidson, 2015; Kabiru et al., 2017). Moreover, statistically significant healthcare systems access effects partially support medical mesocosms (Odum, 1984), which suggests that medical dominance (MD) theory via pattern-matching (Trochim, 2005; Punch, 2014) may illuminate socio-behavioral patterns within spousal violence in households in SSA. Finally, healthcare access effects on cohort differences in VDSV highlight the broad positive social impacts of adequate healthcare, including reducing socioeconomic inequalities in health (Phelan et al., 2010; Quesnel-Vallée et al., 2021), despite known limitations (Great Britain Working Group on Inequalities in Health et al., 1982; Lutfey and Freese, 2005; Frohlich and Abel, 2014).

The increase in predicted marginal probabilities for Other type VDSV across BCs concurs with lower acceptance of SV amongst younger age groups in SSA (ICF, 2012b), despite higher SV incidence potentially confounding the results (UN-WOMEN, 2024).

Research on healthcare systems access and spousal violence in SSA suggests that variations in VDSV may relate to differing exposure to anti-violence interventions at healthcare facilities (Sprague et al., 2016; Hatcher et al., 2019). Similarly, the power difference measure reflects differing women's autonomy in healthcare decisions (Rutstein and Rojas, 2006; Croft et al., 2023); going from pre-1986 to post-1986 birth cohorts, observed VDSV predicted marginal probabilities change across both healthcare systems access and relationship power differences (small, large, and never-married). Additionally, study results emphasize the moderating effect of relationship power difference (UN-WOMEN, 2024).

Despite limitations, research in SSA also concurs that medicine can help reduce social health inequalities, for instance, among the urban poor facing poor health outcomes and limited healthcare access (African Population and Health Research Center, 2002, 2014). First, medical research has increased awareness of social health disparities related to urban–rural and within-urban area differences (Ezeh et al., 2017; Lilford et al., 2017). Moreover, access to maternal healthcare, such as the Output-Based Aid (OBA) Voucher Program, has resulted in fewer home deliveries (Bellows et al., 2012) and lower infant mortality risks (Amendah et al., 2013). However, it correlates with higher spousal violence (Izugbara and Ngilangwa, 2010; Njuki et al., 2012) and increased maternal mortality risks due to inadequate healthcare resources (Ziraba et al., 2009b), as well as the threat of catastrophic healthcare costs (Buigut et al., 2015). Additionally, immunization interventions have not fully resolved inequities in access (Ettarh et al., 2012; Egondi et al., 2015).

Still, interpretations must acknowledge several study limitations. First, household surveys pose several challenges. Spatial interpolation of self-reported data for neighborhood-level measures may exacerbate inaccuracies, underestimating highs and overestimating lows (Van Ham and Manley, 2012; Duncan and Kawachi, 2018). Sampling errors can occur (McNabb, 2020; Olson, 2020) due to the underrepresentation of wealthier households (Wolff, 1987) and imprecision at smaller sub-national levels (ICF International, 2012; Greenwell and Salentine, 2018). Additionally, other errors (McNabb, 2020; Olson, 2020), such as non-response and underreporting (Wolff, 1987; Morelli et al., 2015) and their subsequent effects, such as selection, reporting, and recall biases (Wolff, 1987; O'Donnell and Wagstaff, 2008). To address these issues, the results were adjusted for socioeconomic disparities (Trochim, 2005), and sample weights were applied to account for non-response and underreporting (Lee and Forthofer, 2006). Recent surveys helped mitigate recall and reporting biases (Magadi and Desta, 2011), and control groups of unaffected women were included to reduce selection bias (Lee and Forthofer, 2006). Nationally representative household surveys remain crucial in countries with limited vital statistics (Joubert et al., 2012; Ye et al., 2012). However, implementation challenges, such as poor weather and limited resource availability (Iburg et al., 2001; Greenwell and Salentine, 2018), can cause delays between surveys, impacting pooled analyses (Iburg et al., 2001; Croft et al., 2023); predefined data collection periods, DHS Phase Identifiers, were established to help manage these delays (UNICEF, 2015; Greenwell and Salentine, 2018).

Second, outside low-and-middle-income countries, SV interventions target both public health and legal redress systems (Rizo and Macy, 2011; Prosman et al., 2014; Huntley et al., 2019; Satyen et al., 2019), within SSA; however, policy-makers, partly due to reported SV-accepting social norm barriers in legal redress systems (Odero et al., 2014; Mannell et al., 2016; Gillum et al., 2018), primarily advance public health SV interventions (Joyner, 2013), which may bias study findings (Shadish et al., 2001; Trochim, 2005). During the Coronavirus disease (COVID-19) lockdowns, for instance, SV victims increased, but healthcare systems worldwide prioritized COVID-19 (Akudolu et al., 2023; UN-WOMEN, 2024). Some post-pandemic studies suggested roles for alternative SV support from informal neighborhood social networks, multi-sectoral government support, and non-governmental organizations (Kibe et al., 2020; Wood et al., 2022a; Engdawork et al., 2024). Other studies, however, advocated for healthcare systems support, recommending health caregiver re-training (Wood et al., 2022b) and expanded allied health professionals' involvement, such as including radiologists (Matoori et al., 2021).

Third, to mitigate the risk of social desirability bias in discussions about marriage and sexuality (Ezenweke, 2016; Mannell et al., 2016), a behavioral measure was employed to capture SV perceptions (Mackie et al., 2015). Additionally, to mitigate survey data limitations, several constructed or proxy measures, such as healthcare systems access, were incorporated (Croft et al., 2023). Moreover, the measurement framework used, drawn from macro-meso-micro analogy (Mackie et al., 2015), constitutes multiple social theories but limited accounting for between-theory mediation effects (Ransome, 2010); predictive margins estimated socio-ecological theory hypothesized macro, mezzo, micro, and exosystem interaction effects (Wright, 1997; Bronfenbrenner, 2005), achieving reproducibility (Trochim, 2005; Punch, 2014) but compromising other conditional interaction possibilities (Ransome, 2010; Galster, 2012; Dahlgren and Whitehead, 2021). Moreover, pattern-matching Leventhal's “common-sense-model” theory, which utilizes cyclic illness perception measures (Leventhal et al., 2016), VDSV1, VDSV2, and VDSV3, was derived from related measures, SV experience, help-seeking for SV, and SV accepting attitudes, a suboptimal approach due to internal validity risks (Shadish et al., 2001; Trochim, 2005). Overall, non-standardized measures can undermine plausibility testing, aka. related research evidence comparability (Pearlin, 1989; Cohen et al., 1995; Wright, 1997), a critical factor for establishing strong causal associations (Bonita et al., 2006; Oakes and Kaufman, 2017); evidence on challenges associated with different conceptualizations of stress (Pearlin, 1989; Cohen et al., 1995) and social class (Wright, 1997) corroborate this. Nonetheless, differing trends in VDSV patterns 1 and 2 counter VDSV 3, suggesting that alternative measures may provide valuable insights, augmenting existing survey practices, specifically concerning VDSV pattern 3 (Croft et al., 2023).

Furthermore, the question arises, why, despite known limitations (Odum, 1984; Carpenter, 1996), interrogate esoteric human behavior through mesocosms of equivalent better-understood human behavior? For theory and practice, partially significant healthcare system access modification results imply fragmentary multi-contextual importance of healthcare mesocosms. A contra-mesocosm argument of Parsons' teacher–student and physician–patient interactions' mesocosms (Dillon, 2020) maybe that, while current social systemic influence, including systemic interactions influence, is assumed, unique preceding social system influence is ignored, such as across gender, race, and social class (Zgourides and Zgourides, 2000). System stativity and stability are also incorrectly assumed, and person-to-person differences are ignored (Zgourides and Zgourides, 2000). Moreover, a contra-mesocosm argument of the physician–patient and spouse–partner relationships' mesocosm may be that, while spouse–partner power-asymmetry is primarily personal, physician–patient power-asymmetry is mainly professional (Dillon, 2020), as such, equal power asymmetry may be inconsonant.

Contra-mesocosm debates also incorporate pro-microcosm and pro-macrocosm analogy debates. Pro-microcosm analogy debates suggest using smaller social context studies to explicate larger contexts or single-case studies to explicate multi-person case studies. Specifically, pro-microcosm debates include bridging social process expectations and reality (Auyero, 1999) and transcending social attitudes to illustrate the social phenomenon under study (LaPiere, 1934), such as through participatory observation (Hochschild, 1997; Olson, 2020). Pro-macrocosm analogy debates postulate using larger social contexts to explicate smaller contexts or many to explain one, such as multi-country studies. Specifically, pro-macrocosm debates include relatively low costs for comprehensive large-number studies (Lamont and Swidler, 2014) and within-study error correction methods (Jerolmack and Khan, 2014; Lamont and Swidler, 2014) for misreporting (Jerolmack and Khan, 2014) or bias (Lamont and Swidler, 2014), cross-disciplinary transference (Lamont and Swidler, 2014).

Pro-mesocosm debates encompass contra-microcosm and contra-macrocosm analogy debates. Contra-microcosm analogy debates include limited generalizability of study findings (Weiss, 2004; Small, 2009), positionality bias (McCorkel and Myers, 2003; Takacs, 2003), and overdependence on master narratives (McCorkel and Myers, 2003) for unique stories (Katz, 1997). Contra-macrocosm analogy debates include objectivity rather than subjectivity, implying limited in-depth subject study (Lamont and Swidler, 2014), outlier exclusion (Lamont and Swidler, 2014), and methodological individualism (Heath, 2005), as opposed to collectivism, where social groups and structures primarily explain individuals. However, results illustrate mesocosm applicability, MD, to understanding social systems' influence on VDSV within households in SSA, which also endures across different VDSV estimation methods, postulating strong mesocosm effects.

Study results advocate healthcare systems' approaches to social problems (Conrad and Schneider, 1992; Ityo, 2010; UN-WOMEN, 2024) in SSA, specifically healthcare systems' theory-based approaches. Healthcare system access effects indicate VDSV sensitivity to macro-societal changes, suggesting roles for macro-societal policy (UN-WOMEN, 2024). Notably, the data indicate shifts in cohort differences correspond with significant reproductive health policy milestones, such as the 1984 Mexico International Conference on Population Development (United Nations, 1984). Findings emphasize comprehensive yet localized adoption of macro-societal policies, such as supporting evidence-based best practices in the ongoing implementation of The Maputo Protocol of 1995 (Ayeni, 2016; Nyarko, 2016; Mutuku, 2023; Adesina, 2024). Future research may explore the implications of such findings today, particularly in light of donor healthcare funding cuts in developing countries (Bosire, 2025; World Health Organization, 2025).

## Data Availability

Publicly available datasets were analyzed in this study. This data can be found here: https://dhsprogram.com/data/available-datasets.cfm.
